# Cation-induced ring-opening and oxidation reaction of photoreluctant spirooxazine–quinolizinium conjugates

**DOI:** 10.3762/bjoc.16.82

**Published:** 2020-05-05

**Authors:** Phil M Pithan, Sören Steup, Heiko Ihmels

**Affiliations:** 1 Department of Chemistry and Biology, University of Siegen and Center of Micro- and Nanochemistry and Engineering (Cμ), Adolf-Reichwein-Str. 2, 57068 Siegen, Germany

**Keywords:** oxazole, oxidation, quinolizinium, spirooxazines, styryl dyes

## Abstract

Two new spiroindolinonaphthoxazine derivatives with an electron-accepting styrylquinolizinium or styrylcoralyne unit, respectively, were synthesized, and the influence of such an arylvinyl substituent on the chemical and photochemical properties of the compounds was investigated. Specifically, these spirooxazines turned out to be resistant towards the photoinduced merocyanine formation, and the irradiation with light mainly led to photodegradation of the substrates. However, it was shown by colorimetric and fluorimetric screening assays as well as by detailed NMR spectroscopic and mass spectrometric studies that the addition of particular metal ions (Cu^2+^, Fe^3+^, and to a certain extent Hg^2+^) initially induced a ring-opening reaction that was irreversibly followed by a fast ring closure–deprotonation–oxidation sequence to give styryl-substituted naphthoxazole derivatives as the products quantitatively. For the quinolizinium-substituted spirooxazine derivative, the formation of the respective oxidation product caused the development of a broad absorption band between 425 nm and 500 nm and a new emission band at λ_fl_ = 628 nm, so that it may be employed as a selective chemosensor or chemodosimeter for the colorimetric and fluorimetric detection of Cu^2+^ and Fe^3+^.

## Introduction

**Scheme 1:**
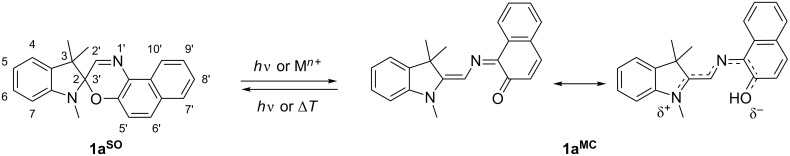
Photo- or cation-induced ring-opening reaction of spirooxazine **1a^SO^**; M*^n^*^+^ = Pb^2+^, La^3+^, Eu^3+^, Tb^3+^ [17].

Spiropyrans and spirooxazines are exemplary photochromic compounds that have gained great attention in the last decades because they allow to reversibly alter and control the physical and chemical properties of functional compounds and materials through the irradiation with light [1–14]. These classes of compounds possess advantageous properties, such as good synthetic accessibility, which allows a broad range of structural modifications and high quantum yields of the photoreaction [12,15–16]. The photochromism is based on a reversible electrocyclic reaction that proceeds through the UV light-induced cleavage of the C2’–O bond of the closed, colorless form to give the colored, metastable merocyanine form, whereas the back reaction can be initiated thermally or by the irradiation with visible light, as is exemplarily shown for the spiroindolinonaphthoxazine **1a** (Scheme 1) [15].

The merocyanine form **1a^MC^** essentially possesses a planar structure, and because of the extended π system, the absorption maximum is significantly red-shifted in comparison to the closed form (e.g., **1a^SO^**: λ_abs_ = 317 nm, **1a^MC^**: λ_abs_ = 602 nm, in MeCN) [16]. In some instances, the ring-opening reaction may also be induced in the dark through the addition of particular metal cations that are able to coordinate to the phenolate oxygen atom and/or other additional coordinating atoms and substituents that have been introduced into the structure of the spirooxazine and spiropyran derivatives [17–26]. Regardless of whether the ring-opening reaction is induced by cations or irradiation, the coordination of metal ions to the substrate usually results in a negative photochromism, i.e., a blue shift of the absorption maximum of the merocyanine form [5,19,27–29], and increased stability of the latter towards the thermal back reaction [21,28,30–34]. In this context, the complexation of metal cations has also been exploited to utilize this compound class for the development of photochromic chemosensors that allow the colorimetric or fluorimetric detection of particular analytes [5,29,35–41]. Furthermore, studies of such spiro derivatives [16,42–43], specifically spiroindolinonaphthoxazines [44–47], have shown that the position of the absorption maximum and the quantum yields or the rate constants of the forward and reverse reaction also depend largely on the solvent polarity as well as on the position and the nature of the substituents either at the indoline or the chromene unit. Surprisingly, there are only a few examples reported for arylvinyl-substituted spirooxazine derivatives, although they possess some interesting photophysical and photochromic properties [16,48–50]. Specifically, the introduction of an arylvinyl [50] or a cyanovinyl substituent [16] causes a red shift of the absorption due to an extended π system or an intramolecular charge transfer (ICT). This property may allow to use visible light with longer wavelength to induce the ring-opening reaction, which would be favorable, e.g., for biological applications. The stilbene etheno bridge, however, may also lead to an intricate photochromic behavior as it can undergo isomerization and cyclization reactions. For example, the irradiation of a 5’-naphthylvinyl-substituted spirooxazine derivative led not only to a ring opening of the oxazine unit but also to an irreversible loss of the photochromic properties, resulting from a cyclization–oxidation reaction under aerobic conditions [51–53]. In this context, we were interested in the effect of a positively charged arylvinyl substituent on the photochromic properties of the spironaphthoxazine. For this purpose, we chose the quinolizinium ion as the cationic aryl substituent because it has been shown that derivatives thereof have a great potential to serve as functional units in DNA-binding ligands [54], fluorescent dyes and chemosensors [55–59]. Furthermore, we have already demonstrated that styryl-substituted quinolizinium derivatives exhibit ideal photophysical and DNA-binding properties to be used as DNA-sensitive fluorescent probes in cell imaging [60–62] or as photoswitchable DNA ligands [63]. Therefore, we were interested in a combination of the photochromic properties of the spirooxazine moiety with the advantageous photophysical and biological properties of the quinolizinium ion to develop photoswitchable functional quinolizinium derivatives. With this goal in mind, we synthesized and investigated the spirooxazine–quinolizinium conjugates **3a** and **3b**. But much to our surprise, our studies revealed that these derivatives are among the rare examples of photoreluctant spirooxazine derivatives with regard to the electrocyclic ring opening, and they could not be employed as molecular photoswitches. At the same time, however, we discovered that the addition of Cu^2+^ or Fe^3+^ to the conjugates **3a** and **3b**, respectively, led to the fast and quantitative formation of indolinonaphthoxazoles. Herein, we present the synthesis of, and investigation on the conjugates **3a** and **3b**, as well as mechanistic studies on the metal ion-induced formation of the derivatives **4a** and **4b**, along with the pertinent spectroscopic data of the products.

## Results and Discussion

### Synthesis

**Scheme 2:**
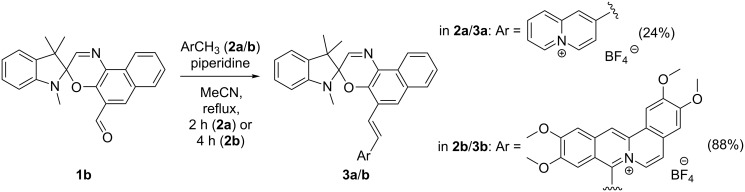
Synthesis of the spirooxazine–quinolizinium conjugates **3a** and **3b**.

The spirooxazine–quinolizinium conjugates **3a** and **3b** were synthesized by the base-catalyzed reaction [60,62,64–65] of 2-methylquinolizinium (**2a**) [66–67] or coralyne (**2b**) [68] with the 5’-formyl-substituted spirooxazine **1b** [48–49,69] (Scheme 2 and Scheme S1, Supporting Information File 1). The structures of the new compounds **3a** and **3b** were confirmed by NMR spectroscopy (^1^H, ^13^C, COSY, HSQC, HMBC), mass spectrometry (ESIMS), and elemental analysis.

### Photophysical and photochemical properties

The absorption spectra of **3a** and **3b** exhibit the characteristic long-wavelength absorption bands of the styryl-substituted quinolizinium or the coralyne chromophore, respectively, with maxima at 386 nm (**3a**) or 437 nm (**3b**) in MeCN (Figure S1A, Supporting Information File 1) [60–61]. Notably, the photoinduced electrocyclic ring-opening reaction of these substrates to give the respective merocyanine forms could not be accomplished. Specifically, the irradiation of the substrates **3a** and **3b** under aerobic conditions at 315 nm or 360 nm did not lead to significant changes in the absorption spectra. However, the irradiation of **3a** at 420 nm caused a decrease of the absorption over the course of several hours, which pointed towards a gradual photodegradation of the substrate (Figure S2A, Supporting Information File 1). For **3b**, the irradiation at 420 nm led to a decrease of the absorption and a bathochromic shift of the long-wavelength absorption band, and after an irradiation time of 60 min, the spectrum showed distinct absorption bands with maxima at 463 nm and 325 nm (Figure S2B, Supporting Information File 1), which was in agreement with our recent finding that 8-styryl-substituted coralyne derivatives undergo a photocyclization–oxidation reaction upon irradiation under aerobic conditions to form pyrroloquinolizinium derivatives (Scheme S2, Supporting Information File 1) [70].

### Reaction with metal ions

**Figure 1:**
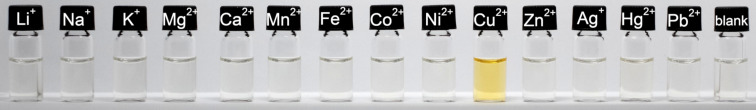
Colors of the solutions resulting from the addition of metal ions (*c* = 50 µM) to derivative **3a** (*c* = 20 µM in MeCN); *t* = 1 h.

As the substrates **3a** and **3b** turned out to be resistant towards the photoinduced merocyanine formation, we tested whether the ring-opening reaction of **3a** and **3b** may be achieved by the treatment with metal ions as this has been shown for other derivates already [17–26]. Since the color change upon conversion of the substrate can usually be seen by the naked eye, a colorimetric screening was performed with a series of different metal cations to check their propensity to induce a reaction of **3a** and **3b**. Notably, within the series of representative alkali and earth alkali ions (Li^+^, Na^+^, K^+^, Mg^2+^, Ca^2+^) and transition metal cations (Mn^2+^, Fe^2+^, Co^2+^, Ni^2+^, Cu^2+^, Zn^2+^, Ag^+^, Hg^2+^, Pb^2+^), only the addition of Cu^2+^ caused a significant coloration of the initially colorless solution of **3a** (*c* = 20 µM) in MeCN (Figure 1). In addition, the solution containing Hg^2+^ also showed a faint coloration.

**Figure 2:**
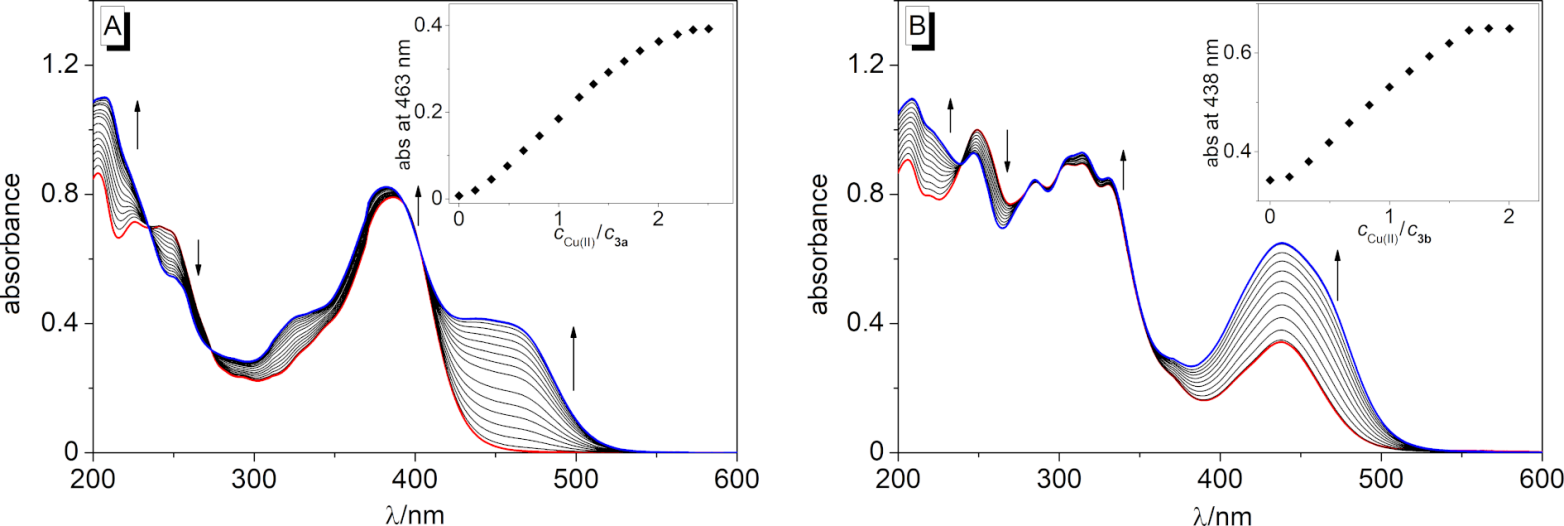
Spectrophotometric titration of **3a** (A) and **3b** (B) (*c* = 20 µM) with Cu(BF_4_)_2_ (*c* = 2.44 mM) in MeCN. Red: Spectra of solutions of **3a** and **3b** in the absence of metal ions; blue: spectra at the end of the titrations. The arrows indicate the changes of absorption upon the addition of Cu^2+^. Insets: Plot of the absorbance vs *c*_Cu(II)_/*c***_3a_**_/_**_b_**.

For **3b**, the addition of Cu^2+^ just led to a distinct color intensification of the already yellow solution, whereas the addition of all other metal ions from the employed series did not induce any apparent changes (Figure S3, Supporting Information File 1); however, the latter effect is not conclusive due to the inherent color of the solution that may obscure smaller color changes. For a detailed investigation, the changes of the absorbance upon the addition of Cu^2+^ to the spirooxazine conjugates **3a** and **3b** were followed spectrophotometrically (Figure 2). The addition of Cu^2+^ to a solution of **3a** caused a slight blue shift of the absorption maximum (Δλ = 4 nm) and the formation of a very broad additional absorption band between 425 nm and 500 nm (Figure 2A and Table 1), whereas the titration of Cu^2+^ to a solution of **3b** caused only a significant increase of the initial absorption. In effect, the position of the long-wavelength absorption maximum at λ_abs_ = 438 nm remained unchanged, although the absorption band also showed a weak red-shifted shoulder at the end of the titration (Figure 2B). Remarkably, the plots of the absorption vs *c*_Cu(II)_/*c***_3a_**_/_**_b_** exhibited an inflection point at ca. 0.5 equiv of Cu^2+^, which may indicate that the final product is formed at least in a two-step mechanism (insets in Figure 2). After the addition of ca. 2.5 equiv of Cu^2+^ for **3a** or 2.0 equiv for **3b**, no further changes in the absorption spectra were detected. For **3a**, further monitoring by absorption spectroscopy revealed that higher Cu^2+^ concentrations led to an increase of the reaction rate. However, at a spirooxazine concentration of *c* = 20 µM, slightly more than 2.0 equiv of Cu^2+^ were required for a complete conversion (Figure S4, Supporting Information File 1). At the same time, the reaction was already completed after ca. 25 min in the presence of 3.0 equiv of Cu^2+^ (Figure S4B, Supporting Information File 1). In contrast, the reaction in the presence of 3.0 equiv of Hg^2+^ was very slow and not complete even after 18 h (Figure S5, Supporting Information File 1).

**Table 1:** Absorption and emission properties of the derivatives **3a**, **3b**, **4a**, and **4b**.

Derivative	λ_abs_^a^/nm	lg ε^b^	λ_fl_^c^/nm	Φ_fl_^d^/10^−2^

**3a**	386	4.60	524^e^	<0.1^f^
**3b**	438	4.23	481^e^	0.7^f^
**4a**^g^	382	4.61	628^h^	12^i^
**4b**^g^	438	4.51	481^e^	n.d.^j^

^a^Long-wavelength absorption maximum; *c* = 20 µM. ^b^Molar extinction coefficient in cm^–1^⋅M^–1^. ^c^Fluorescence emission maximum; *c* = 5 µM. ^d^Estimated error for the fluorescence quantum yields: ±10%. ^e^λ_ex_ = 400 nm. ^f^Fluorescence quantum yield relative to Coumarin 307 (Φ_fl_ = 0.58) [71]. ^g^Data determined from solutions of **3a** and **3b** 1 h after the addition of 3 equiv of Cu^2+^. ^h^λ_ex_ = 470 nm. ^i^Fluorescence quantum yield relative to rhodamine 6G (Φ_fl_ = 0.95) [72]. ^j^Not determined.

**Figure 3:**
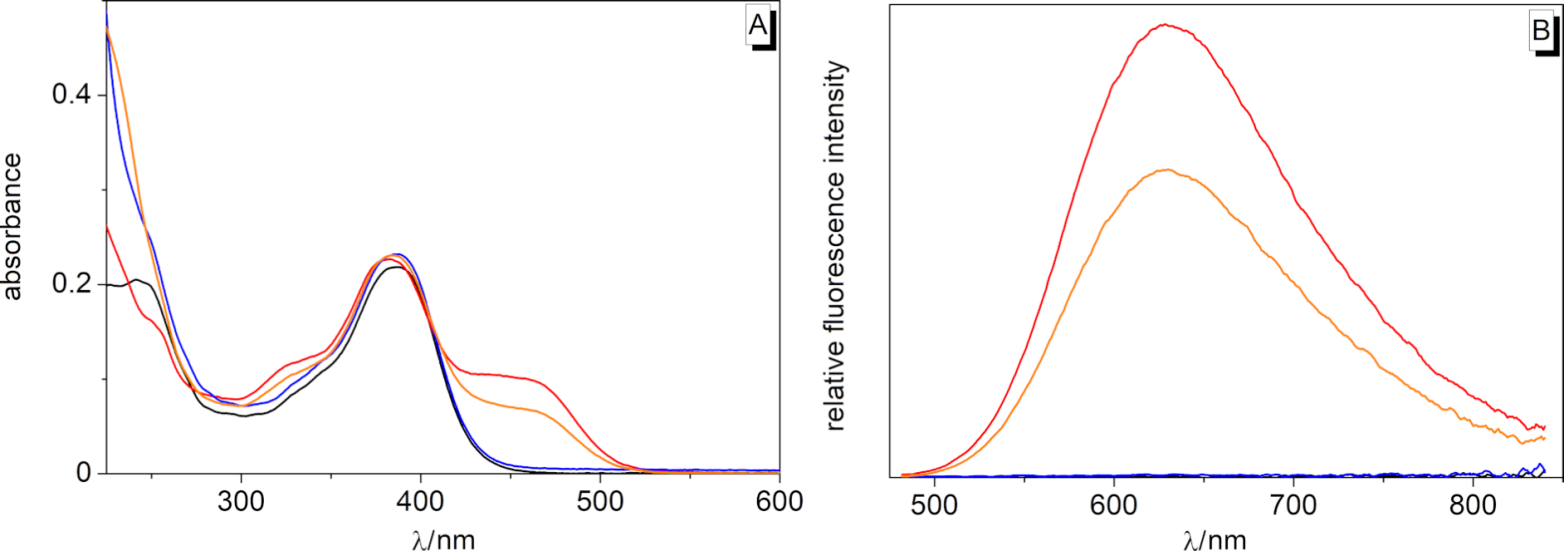
Absorption (A) and fluorescence spectrum (B) of **3a** in MeCN (*c* = 5 µM) in the absence (black) and in the presence of metal ions ca. 1 h after the addition. Red: Cu^2+^ (*c* = 15 µM), orange: Hg^2+^ (*c* = 100 µM), blue: Li^+^, Na^+^, K^+^, Mg^2+^, Ca^2+^, Mn^2+^, Fe^2+^, Co^2+^, Ni^2+^, Zn^2+^, Ag^+^, and Pb^2+^ (*c* = 100 µM each); λ_ex_ = 470 nm.

**Figure 4:**
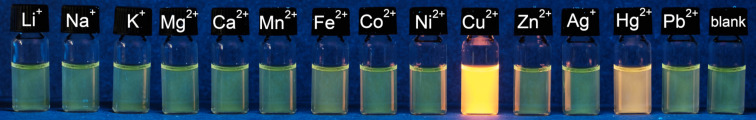
Emission colors of solutions resulting from the addition of metal ions (*c* = 50 µM) to derivative **3a** (*c* = 20 µM in MeCN); *t* = 1 h; λ_ex_ = 366 nm.

The compound **3a** is essentially nonfluorescent in solution. The addition of 3.0 equiv of Cu^2+^, however, led to the development of a new, broad emission band at λ_fl_ = 628 nm (Φ_fl_ = 0.12 relative to rhodamine 6G [72], Figure 3B and Table 1). As supported by the corresponding excitation spectrum (Figure S1B, Supporting Information File 1), this emission most likely originated from the excitation of the new absorption band between 425 nm and 500 nm (Figure 3A) and can also be seen by the naked eye under UV light (Figure 4). It should be noted, however, that the excitation at lower wavelengths led to the development of additional weak emission bands, likely caused by conformational changes in higher excited states, a phenomenon that was not further assessed in this study. Because of the much lower reaction rate, even a 20-fold excess of Hg^2+^ led to a less pronounced orange-colored fluorescence at ca. 1 h after the addition. All the other tested metal ions (see above) did not cause a change of the absorption and emission. The coralyne–spirooxazine conjugate **3b** was weakly fluorescent upon the excitation at λ_ex_ = 400 nm, with a broad emission band at λ_fl_ = 481 nm (Φ_fl_ = 0.007 relative to coumarin 307 [71], Figure S1A, Supporting Information File 1 and Table 1), but the shift and intensity of the emission band did not change upon the addition of Cu^2+^.

**Scheme 3:**
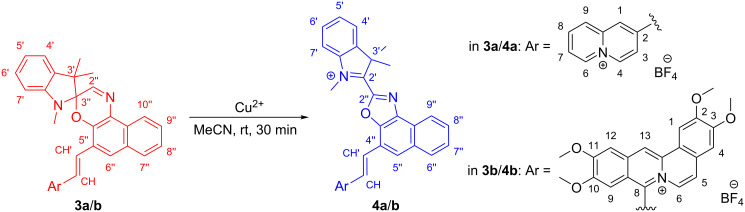
Cu^2+^-induced formation of the oxazole derivatives **4a** and **4b**.

**Figure 5:**
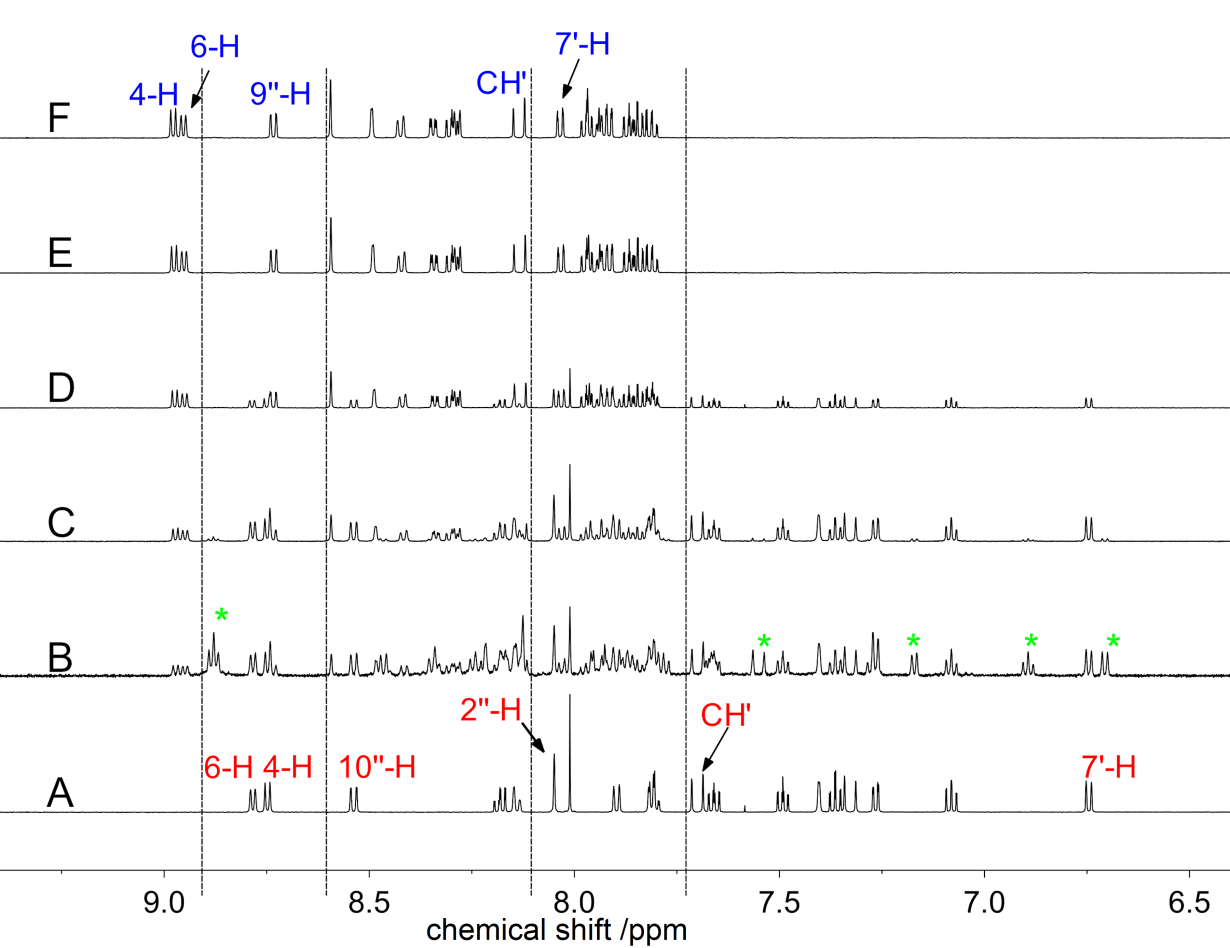
^1^H NMR spectra (600 MHz, 6.4–9.4 ppm) of **3a** (*c* = 2.0 mM) in the absence (A) and in the presence (B–F) of Cu^2+^ (B: 0.50 mM, C: 1.0 mM, D: 2.0 mM, E: 3.0 mM, F: 4.0 mM) in CD_3_CN (cf. Scheme 3). The green asterisks highlight the clearly distinguishable signals of the third signal set of a reaction intermediate (B) in addition to the signal sets of **3a** (A) and **4a** (F).

The reaction of **3a** upon the addition of Cu^2+^ was also investigated by ^1^H NMR spectroscopy in order to obtain more insight into the mechanism (Figure 5 and Figure S6, Supporting Information File 1). The ^1^H NMR spectrum of **3a** showed 19 protons in the aromatic region (>6.5 ppm), with the characteristic signals of the quinolizinium unit at δ_H_ = 8.78 (d, 6-H) and 8.74 (d, 4-H) and of the spirooxazine moiety at δ_H_ = 8.53 (d, 10’’-H), 8.05 (s, 2’’-H), 6.74 (d, 7’-H), 2.76 (s, NMe), 1.44 (s, 3’-Me), and 1.38 (s, 3’-Me, Figure 5A and Figure S6A, Supporting Information File 1). With increasing copper ion concentration, the signal intensities decreased, and a new set of signals developed, which only consisted of 18 protons in the aromatic region (Figure 5B–F and Figure S6B–F, Supporting Information File 1). Notably, a third set of signals was detected when employing copper-to-ligand ratios of 0.25 and 0.50 (Figure 5B,C and Figure S6B,C, Supporting Information File 1, marked with green asterisks). While the signals in the aromatic region could not be dissected unambiguously from the other signal sets, the three singlets in the aliphatic region at δ_H_ = 2.87 (s, NMe), 1.56 (s, 3’-Me), and 1.02 (s, 3’-Me, Figure S6B,C, Supporting Information File 1) most likely corresponded to a spirooxazine species in its closed form, as indicated by the characteristic chemical shifts. The final product of the reaction was identified by additional NMR spectroscopic investigations (^13^C, COSY, HSQC, HMBC) and mass spectrometric analysis (ESI) as the oxazole derivative **4a** (Scheme 3). The latter analysis confirmed the loss of one hydrogen atom from the substrate **3a** at C2’’ (*m*/*z* = 241 [M − 2BF_4_]^2+^, 568 [M − BF_4_]^+^). The ^1^H NMR signals of **4a** exhibited a significant downfield shift in comparison to the signals of **3a**. Specifically, the spectrum showed signals at δ_H_ = 8.98 (d, 4-H), 8.96 (d, 6-H), 8.72 (d, 9’’-H), 8.04 (d, 7’-H), 4.81 (s, N^+^Me), and 2.11 (s, 2 × 3’-Me). Moreover, the ^13^C NMR signal of C2’ also exhibited a downfield shift from δ_C_ = 100.7 to 170.8. Thereby, both the ^1^H and the ^13^C NMR shifts of the indolinium–naphthoxazole unit were in good agreement with the reported ones of similar derivatives [17,73–76]. At the same time, the addition of 2 equiv of Cu^2+^ to **3b** also resulted in the formation of the corresponding oxazole derivative **4b** (Scheme 3), as shown by ^1^H NMR spectroscopic and mass spectrometric analysis. Additionally, the NMR spectroscopic analysis (^1^H and H,H-COSY) of a solution of **4a** in MeCN after the addition of water revealed the formation of the corresponding hydroxy-substituted derivative **5**, resulting from the nucleophilic addition at the carbon atom C2’ (Scheme S3, Supporting Information File 1). This was clearly evidenced by the characteristic ^1^H NMR signals [74,77] at δ_H_ = 6.69 (d, 7’-H), 3.23 (s, NMe), 1.48 (s, 3’-Me), and 0.93 (s, 3’-Me) that are again significantly shifted upfield in comparison to the ones of **4a**.

**Figure 6:**
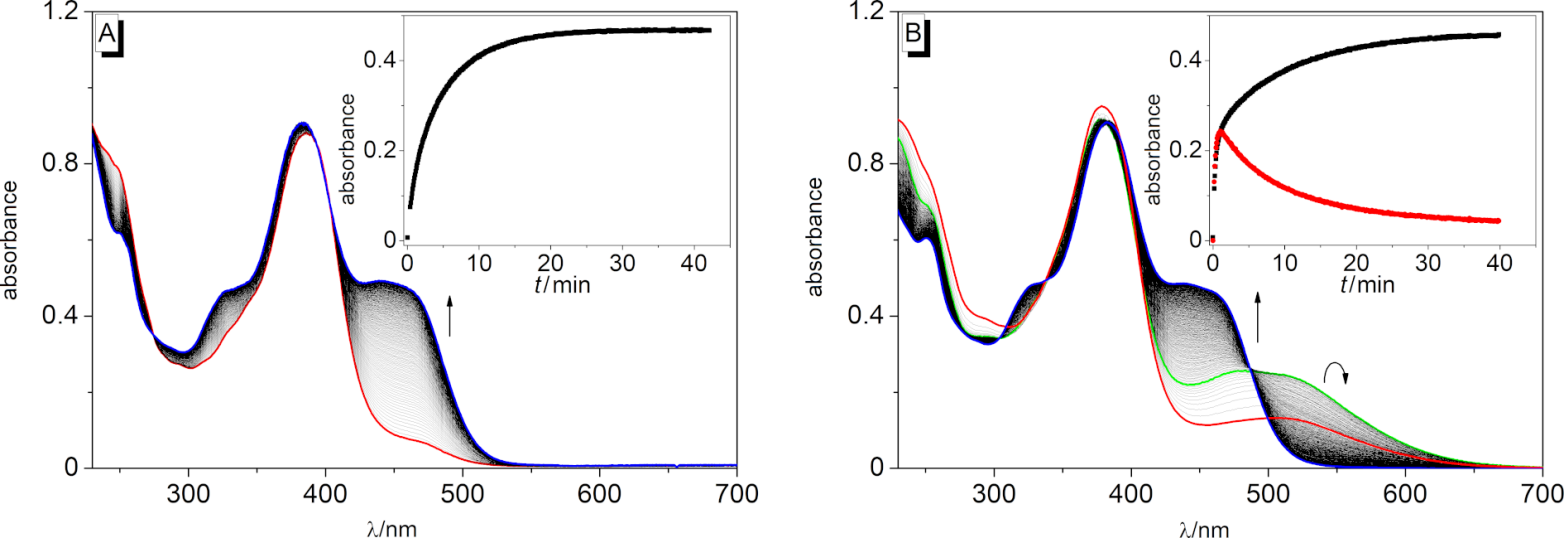
Spectral changes of **3a** (*c* = 20 µM) upon the addition of Cu^2+^ (A) and Fe^3+^ (B) (*c*_M+_ = 60 µM) in MeCN. The arrows indicate the changes of the absorption during the reaction. Red: 25 s (A) or 10 s (B) after the addition, green: 60 s, blue: 40 min. Inset: Plot of the absorption at 463 nm (black) and 515 nm (red) vs the reaction time *t*.

It has already been shown that spirooxazines may react in certain cases to oxazole derivatives. However, depending on the employed conditions, different mechanisms for the formation of these oxidation products have been proposed [17,73–80]. For example, Fedorova et al. have shown that after the cation-induced ring opening of spirooxazine derivatives, the respective merocyanine forms undergo a slow oxidation to the corresponding oxazoles in the dark only under aerobic conditions [17,80]. The reaction was apparently promoted by the complexation of the metal ions as such oxazole derivatives were originally only observed as intermediates during the photooxidation of spirooxazines in aerated solutions [78,81]. In another case, Uznanski et al. demonstrated that in the presence of silver(I) or gold(III) ions, the spironaphthoxazine **1a^SO^** or the corresponding 5-chloro-substituted analog underwent thermal–oxidative degradation even under an inert gas atmosphere in the dark [74–75]. Based on the formation of silver or gold nanoparticles during the reaction, the authors proposed a two-step electron transfer from the merocyanine to the metal cations, which acted as electron acceptors. Similarly, Malatesta et al. found that the thermal dark reaction of spironaphthoxazines in the presence of a suitable electron acceptor, such as 7,7,8,8-tetracyanoquinodimethane, gave the corresponding naphthoxazole derivatives as a result of electron-transfer processes [76]. To compare our results with the literature data, we performed a corresponding control experiment under the exclusion of oxygen. Thus, upon the addition of Cu^2+^ to **3a**, the oxazole **4a** was formed in the same manner as under aerobic conditions, indicating an electron transfer from a reaction intermediate to the copper ions. In addition, we tested whether **4a** may also be formed upon the addition of the previously not employed Fe^3+^ ion as this also acts as a strong electron acceptor (Figure 6B). Indeed, the addition of Fe^3+^ to **3a** resulted in the formation of **4a**, as indicated by the characteristic absorption band between 425 nm and 500 nm (Figure 6B, blue spectrum), although the reaction proceeded slightly slower in comparison to the addition of Cu^2+^ under the same conditions (Figure 6A). Remarkably, in the presence of Fe^3+^, the initial development of a broad absorption band between 500 nm and 650 nm was also observed, which again gradually disappeared during the course of the reaction. These observations pointed towards the formation of the red-colored, open merocyanine form as a very reactive intermediate during the reaction (see discussion below).

**Scheme 4:**
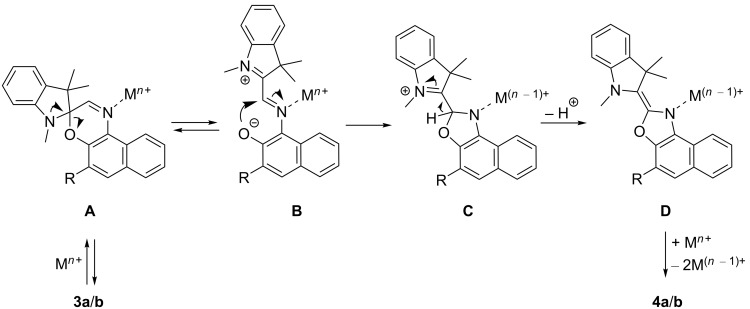
Proposed mechanism for the formation of the oxazole derivatives **4a** and **4b** (cf. Scheme 3); M*^n^*^+^ = Cu^2+^, Fe^3+^, Hg^2+^.

According to the concurrent assumption of the proposed mechanisms of the cation-induced ring opening of spirooxazines (see above), the metal cation initially coordinated to the phenolate oxygen of the merocyanine. Based on the possible transoid structures, it has already been discussed in the literature whether metal cations are coordinated in a monodentate fashion to the phenolate oxygen atom or rather in a bidentate fashion both to the phenolate oxygen and the imine nitrogen atoms of the open merocyanine form [25,28]. Furthermore, density functional theory (DFT) calculations have shown that depending on the nature of the metal ion, the adopted position relative to the nitrogen and oxygen atoms may vary [82]. In **3a** and **3b**, the electron density of the phenolate oxygen atom was significantly reduced due to the direct conjugation with the strong electron-accepting styrylquinolinizium unit at the neighboring carbon atom C5’’. At the same time, imine groups also have a high binding affinity to Cu^2+^ [83–85] and Fe^3+^ [86–87] so that we propose the initial coordination of the metal cation mainly to the imine nitrogen atom of the spirooxazine group in the course of the reaction (Scheme 4, **A**). Moreover, this explanation is in agreement with the occurrence of the additional set of signals at small copper-to-ligand ratios (Figure 5B,C and Figure S6B,C, Supporting Information File 1, marked with green asterisks) and with the course of the spectrophotometric titration (Figure 2), thus indicating that the initial coordination of the copper ion to the closed form of the spirooxazine was the rate-determining step of the reaction. And even though the NMR spectra (Figure 5) did not show any signals at δ_H_ > 9.5 ppm that would correspond to 2’’-H of the open merocyanine form [17], we still presume that it was formed after the Cu^2+^-induced ring-opening reaction, but the following processes were too fast so that the intermediate **B** could not be detected by NMR spectroscopy (Scheme 4). Additionally, the development of the absorption spectra in the presence of Fe^3+^ revealed a short-lived absorption band between 500 and 650 nm, which supports this hypothesis and suggests that the processes have different rate constants in the presence of Cu^2+^ or Fe^3+^. We finally propose that the following reaction steps, i.e., the ring closure, deprotonation, and electron transfer to a second metal cation (Scheme 4, **C** and **D**), were relatively fast processes, resulting in the formation of the corresponding oxidation products **4a** and **4b**. Considering this proposed mechanism (Scheme 4), the lack of reaction in the presence of metal cations other than Cu^2+^ or Fe^3+^ or the relatively slow reaction upon the addition of Hg^2+^ may have been caused by the low propensity of these cations to coordinate to the imine functionality and/or the inability to be involved in the subsequent electron transfer.

## Conclusion

The spirooxazine–quinolizinium conjugates **3a** and **3b** were synthesized by a base-catalyzed Knoevenagel reaction from the readily available 5-formyl-substituted spirooxazine **1b**. Due to the introduction of the electron-accepting quinolizinium(ethenyl) substituent, the spirooxazines **3a** and **3b** were photoinert towards the electrocyclic ring opening, thus constituting one of the rare cases of photoreluctant spirooxazines [74]. Nevertheless, studies with a series of different metal cations revealed that the addition of Cu^2+^, Fe^3+^, and, to a certain extent, also Hg^2+^ to **3a** and **3b** induced a fast reaction to give the naphthoxazole products **4a** and **4b** quantitatively. For **3a**, the presence of these metal cations also caused a fluorimetric response due to the developing orange emission at λ_fl_ = 628 nm so that **3a** may be employed as a selective chemosensor or chemodosimeter for the colorimetric and fluorimetric detection of Cu^2+^ and Fe^3+^, respectively. As shown by NMR spectroscopic and mass spectrometric studies, the optical response did not originate from a merocyanine intermediate formed in the cation-induced ring-opening reaction. Instead, the color stemmed from the irreversible formation of the naphthoxazole derivatives **4a** and **4b** after the quantitative oxidation of **3a** and **3b**. Specifically, the Cu^2+^ and Fe^3+^ ions most likely induced a ring opening/ring closing cascade of the spirooxazine unit in **3a** and **3b** including an electron transfer to the respective metal cations. These reaction steps seemed to have different rates in the presence of Cu^2+^ or Fe^3+^, which may even allow the differentiation between those cations as the visible formation of the short-lived merocyanine form only occurred in the presence of Fe^3+^.

## Experimental

### Equipment

NMR spectra were recorded with a Bruker Avance 400 (^1^H: 400 MHz, ^13^C: 100 MHz) at room temperature (approximately 22 °C), with a Jeol ECZ 500 (^1^H: 500 MHz, ^13^C: 125 MHz) at 25 °C, or with a Varian VNMR-S 600 (^1^H: 600 MHz, ^13^C: 150 MHz) at 25 °C. The spectra were processed with the software ACD/NMR Processor Academic Edition (version: 12.02) or MestReNova (version: 12.0.1) and referenced to the respective solvent (DMSO-*d*_6_: δ_H_ = 2.50, δ_C_ = 39.5; CDCl_3_: δ_H_ = 7.27, δ_C_ = 77.0; CD_3_CN: δ_H_ = 1.94, δ_C_ = 1.34). The chemical shifts are given in ppm. Absorption spectra were recorded with a Cary 100 Bio or with an Analytik Jena Specord S 600 spectrophotometer in Hellma quartz cells 110-QS or 114B-QS (10 mm), with baseline correction at 20 °C. Emission spectra were collected with a Cary Eclipse spectrophotometer in Hellma quartz cells 114F-QS (10 mm × 4 mm) at 20 °C. Elemental analyses data were determined with a HEKAtech *EURO*EA combustion analyzer by Mr. Rochus Breuer (Universität Siegen, Organische Chemie I). Mass spectra (ESI) were recorded on a Finnigan LCQ Deca (*U* = 6 kV; working gas: argon; auxiliary gas: nitrogen; temperature of the capillary: 200 °C). The melting points were measured with a BÜCHI 545 (BÜCHI, Flawil, CH) and are uncorrected. Solutions were irradiated with a diode array light apparatus (Atlas Photonics LUMOS 43).

### Materials

The commercially available chemicals were reagent-grade and used without further purification. Absorption and emission spectra were recorded from solutions prepared with spectroscopic grade solvents. Metal salt solutions were prepared from the respective tetrafluoroborate or perchlorate salts at a concentration of *c* = 2.4–2.6 mM in MeCN.

### Methods

The solutions for each measurement were prepared from stock solutions in a suitable solvent (MeCN for **3a**, CHCl_3_ for **3b**; *c* = 1.0 mM). For experiments in different solvents, aliquots of the stock solution were evaporated under a stream of nitrogen and redissolved in the respective solvent.

For spectrometric titrations, aliquots of the spirooxazine solutions were placed into quartz cells and titrated with the metal salt solutions in intervals of 0.15–0.25 equivalents, and after an equilibration time of 3 min absorption spectra were recorded. The titrations were stopped when no more changes were observed in the absorption spectra. All spectrometric titrations were performed at least two times to ensure reproducibility. In general, the absorption spectra were determined in a range between 200 nm and 800 nm and subsequently smoothed with the Origin software function “adjacent-averaging“ (factor of 10).

For the reaction monitoring by absorption spectroscopy, an aliquot of the solutions of Cu(BF_4_)_2_, Fe(ClO_4_)_3_ or Hg(ClO_4_)_2_ was added to a solution of **3a** (*c* = 20 µM) in MeCN to achieve a final concentration of the metal ions of *c* = 20–60 µM. The solutions were mixed vigorously, and absorption spectra were recorded every 5 s.

For the reaction monitoring by NMR spectroscopy, six samples were prepared with a fixed concentration of **3a** (*c* = 2.0 mM). In five of the samples, different amounts of Cu(BF_4_)_2_ were added to obtain Cu^2+^ concentrations of *c* = 0.5, 1.0, 2.0, 3.0 and 4.0 mM. The corresponding aliquots of the stock solutions of **3a** and Cu(BF_4_)_2_ in MeCN were mixed, evaporated, and redissolved in CD_3_CN. The solutions were kept in the dark for 1 h and analyzed.

For the photochemical studies, air-saturated solutions of **3a** and **3b** in MeCN were irradiated with a diode array light apparatus (Atlas Photonics LUMOS 43) at λ_irr_ = 315 nm, 360 nm, or 420 nm in Hellma quartz cells 110-QS (10 mm).

For the detection of emission spectra, the excitation and emission slits were adjusted to 5 nm, the detection speed was 120 nm⋅min^−1^, and the detector voltage was adjusted between 500 V and 600 V, depending on the fluorescence intensity. The emission spectra were smoothed with the implemented moving-average function by a factor of 5. Emission spectra in the range between 600 nm and 850 nm were corrected with an instrument-specific correction curve. The fluorescence quantum yields of the derivatives **3a**, **3b**, and **4a** were determined relative to coumarin 307 (Φ_fl_ = 0.58 in MeCN) [72] or rhodamine 6G (Φ_fl_ = 0.95 in EtOH) [71], according to the established procedures [88–89].

### Synthesis

#### Synthesis of the quinolizinium–spirooxazine conjugates **3a** and **3b**

**(*E*)-2-(2-(1,3,3-Trimethylspiro[indoline-2,3'-naphtho[2,1-*b*][1,4]oxazine]-5'-yl)vinyl)quinolizinium tetrafluoroborate (3a)** [60,62,64–65]: To a solution of 2-methylquinolizinium tetrafluoroborate (**2a**, 116 mg, 500 µmol) and the 5’-formyl-substituted spirooxazine **1b** (214 mg, 600 µmol) in MeCN (15 mL) was added piperidine (42.6 mg, 500 µmol, 49.5 µL) at 80 °C under an argon atmosphere and exclusion of light, and the reaction mixture was stirred under reflux for 4 h. After cooling to rt, the mixture was added dropwise to Et_2_O (300 mL) under vigorous stirring. The precipitate was filtered, washed with Et_2_O (3 × 20 mL) and redissolved in a minimal amount of MeCN. The solution was filtered over a pad of celite and microfiltered (pore size: 0.45 µm). The product **3a** was precipitated by the dropwise addition of the solution to Et_2_O and obtained after recrystallization from MeCN/EtOAc at –25 °C as yellow, microcrystalline solid (83.5 mg, 147 µmol, 24%). mp 275 °C dec. ^1^H NMR (500 MHz, CD_3_CN) δ 1.37 (s, 3H, 3’-Me), 1.43 (s, 3H, 3’-Me), 2.75 (s, 3H, NMe), 6.74 (d, ^3^*J* = 7 Hz, 1H, 7’-H), 7.08 (ddd, ^3^*J* = 8 Hz, ^3^*J* = 8 Hz, ^4^*J* = 1 Hz, 1H, 5’-H), 7.26 (d, ^3^*J* = 7 Hz, 1H, 4’-H), 7.28 (d, ^3^*J* = 17 Hz, 1H, CH), 7.35–7.38 (m, 2H, 1-H, 6’-H), 7.46 (ddd, ^3^*J* = 8 Hz, ^3^*J* = 7 Hz, ^4^*J* = 1 Hz, 1H, 8’’-H), 7.62 (ddd, ^3^*J* = 8 Hz, ^3^*J* = 7 Hz, ^4^*J* = 1 Hz, 1H, 9‘‘-H), 7.65 (d, ^3^*J* = 17 Hz, 1H, CH’), 7.76 (dd, ^3^*J* = 7 Hz, ^4^*J* = 2 Hz, 1H, 3-H), 7.79 (ddd, ^3^*J* = 8 Hz, ^3^*J* = 7 Hz, ^4^*J* = 1 Hz, 1H, 7-H), 7.85 (d, ^3^*J* = 8 Hz, 1H, 7’’-H), 8.00 (s, 1H, 6’’-H), 8.01 (s, 1H, 2’’-H), 8.11 (d, ^3^*J* = 8 Hz, 1H, 9-H), 8.17 (ddd, ^3^*J* = 8 Hz, ^3^*J* = 7 Hz, ^4^*J* = 1 Hz, 1H, 8-H), 8.49 (d, ^3^*J* = 8 Hz, 1H, 10’’-H), 8.72 (d, ^3^*J* = 7 Hz, 1H, 4-H), 8.77 (d, ^3^*J* = 6 Hz, 1H, 6-H); ^13^C NMR (125 MHz, CD_3_CN) δ 20.8 (3’-Me), 25.0 (3’-Me), 30.4 (NMe), 52.6 (C3’), 100.7 (C2’/C3’’), 108.9 (C7’), 120.8 (C3), 121.2 (C5’), 122.5 (C10’’), 122.8 (C4’), 124.0 (C7), 124.2 (C1), 124.5 (C5’’), 124.8 (C10b’’), 126.1 (C8’’), 127.9 (C9), 128.0 (CH), 129.2 (C6’), 129.5 (C7’’), 129.6 (C9’’), 129.7 (C6a’’), 132.0 (C10a’’), 133.7 (C6’’), 135.9 (CH’), 137.1 (C4), 137.3 (C6), 137.6 (C3a’), 138.0 (C8), 144.0 (C4a’’), 144.2 (C9a), 146.8 (C2), 149.0 (C7a’), 152.1 (C2’’); MS–ESI^+^ (*m*/*z*): 482 (100, M − BF_4_); MS–ESI^−^ (*m*/*z*): 656 (79, M + BF_4_), 1225 (100, 2M + BF_4_), 1794 (22, 3M + BF_4_); anal. calcd for C_33_H_28_BF_4_N_3_O·1/3(HBF_4_): C, 66.21; H, 4.77; N, 7.02; found: C, 66.40; H, 4.49; N, 7.33. All values are given as percentages.

**(*E*)-8-(2-(1,3,3-Trimethylspiro[indoline-2,3'-naphtho[2,1-*b*][1,4]oxazine]-5'-yl)vinyl)coralyne tetrafluoroborate (3b)** [60,62,64–65]: To a suspension of coralyne tetrafluoroborate (**2b**, 90.2 mg, 200 µmol) and the 5’-formyl-substituted spirooxazine **1b** (143 mg, 400 µmol) in MeCN (6 mL) was added piperidine (34.1 mg, 400 µmol, 39.6 µL) at 80 °C under an argon atmosphere and exclusion of light, and the reaction mixture was stirred under reflux for 2 h. After cooling to rt, the mixture was added dropwise to Et_2_O (120 mL) under vigorous stirring. The precipitate was filtered and washed with Et_2_O (3 × 20 mL). The product **3b** was obtained as green amorphous solid (138 mg, 175 µmol, 88%). An analytically pure sample was obtained by recrystallization from MeOH at −25 °C. mp 280 °C dec. ^1^H NMR (600 MHz, DMSO-*d*_6_) δ 1.33 (s, 6H, 2 × 3’-Me), 2.79 (s, 3H, NMe), 3.89 (s, 3H, 10-OMe), 4.01 (s, 3H, 3-OMe), 4.12 (s, 3H, 2-OMe), 4.13 (s, 3H, 11-OMe), 6.64 (d, ^3^*J* = 8 Hz, 1H, 7’-H), 6.72–6.75 (m, 1H, 5’-H), 7.05 (ddd, ^3^*J* = 8 Hz, ^3^*J* = 8 Hz, ^4^*J* = 1 Hz, 1H, 6’-H), 7.13 (d, ^3^*J* = 7 Hz, 1H, 4’-H), 7.45 (d, ^3^*J* = 17 Hz, 1H, CH’), 7.50 (s, 1H, 9-H), 7.53–7.56 (m, 1H, 8’’-H), 7.66–7.71 (m, 3H, 4-H, 9’’-H, 12-H), 7.78 (d, ^3^*J* = 8 Hz, 1H, 5-H), 7.79 (d, ^3^*J* = 17 Hz, 1H, CH), 8.00 (d, ^3^*J* = 8 Hz, 1H, 7’’-H), 8.02 (s, 1H, 2’’-H), 8.32 (s, 1H, 1-H), 8.53–8.55 (m, 2H, 6-H, 10’’-H), 8.62 (s, 1H, 6’’-H), 9.69 (s, 1H, 13-H); ^13^C NMR (150 MHz, DMSO-*d*_6_) δ 21.2 (3’-Me), 25.2 (3’-Me), 29.2 (NMe), 51.5 (C3’), 56.1 (3-OMe), 56.1 (10-OMe), 56.4 (2-OMe), 56.7 (11-OMe), 98.8 (C2’/C3’’), 103.9 (C9), 104.8 (C12), 105.0 (C1), 107.0 (C7’), 108.0 (C4), 116.8 (C13), 119.7 (C5’), 119.7 (C13b), 119.8 (CH), 120.6 (C5), 121.3 (C10’’), 121.5 (C8a), 121.6 (C4’), 122.8 (C5’’), 123.2 (C10b’’), 123.9 (C4a), 124.7 (C6), 125.2 (C8’’), 128.0 (C6’), 128.3 (C6a’’), 128.4 (C9’’), 128.5 (C7’’), 129.7 (C6’’), 130.7 (C10a’’), 134.0 (C12a), 134.5 (C13a), 135.1 (C3a’), 136.7 (CH’), 141.9 (C4a’’), 143.4 (C8), 146.8 (C7a’), 151.5 (C2), 152.5 (C2’’), 152.7 (C3), 152.8 (C10), 156.1 (C11); MS–ESI^+^ (*m*/*z*): 702 (100, M − BF_4_); MS–ESI^−^ (*m*/*z*): 1666 (100, 2M + BF_4_); anal. calcd for C_45_H_40_BF_4_N_3_O_5_·0.5 HBF_4_: C, 64.84; H, 4.90; N, 5.04; found: C, 65.03; H, 4.79; N, 5.17. All values are given as percentages.

#### Synthesis of the oxazole derivatives **4a**, **4b** and **5**

Aliquots of the stock solutions of **3a** or **3b** and Cu(BF_4_)_2_ in MeCN were mixed, evaporated, and redissolved in CD_3_CN in order to obtain a concentration of *c***_3a_**_/_**_b_** = 2.0 mM and *c*_Cu(II)_ = 4.0 mM. The solution was kept in the dark for 1 h and then used for the NMR spectroscopic analysis. For the mass spectrometric analysis, aliquots of the stock solutions were redissolved in MeCN in order to obtain concentrations of *c***_3a_**_/_**_b_** = 20 µM and *c*_Cu(II)_ = 60 µM.

**(*E*)-2-(2-(2-(1,3,3-Trimethyl-3*H*-indol-1-ium-2-yl)naphtho[1,2-*d*]oxazol-4-yl)vinyl)quinolizinium (4a):** ^1^H NMR (600 MHz, CD_3_CN) δ 2.11 (s, 6H, 2 × 3’-Me), 4.81 (s, 3H, N^+^Me), 7.80–7.88 (m, 3H, 6’-H, 5’-H, 7’’-H), 7.91–7.98 (m, 4H, 4’-H, 7-H, CH, 8’’-H), 8.04 (d, ^3^*J* = 8 Hz, 1H, 7’-H), 8.13 (d, ^3^*J* = 16 Hz, 1H, CH’), 8.28 (d, ^3^*J* = 8 Hz, 1H, 6’’-H), 8.30 (ddd, ^3^*J* = 8Hz, ^3^*J* = 7 Hz, ^4^*J* = 1 Hz, 1H, 8-H), 8.35 (ddd, ^3^*J* = 7 Hz, ^4^*J* = 2 Hz, 1H, 3-H), 8.43 (d, ^3^*J* = 9 Hz, 1H, 9-H), 8.50 (d, ^4^*J* = 2 Hz, 1H, 1-H), 8.59 (s, 1H, 5’’-H), 8.72 (d, ^3^*J* = 8 Hz, 1H, 9’’-H), 8.96 (d, ^3^*J* = 7 Hz, 1H, 6-H), 8.98 (d, ^3^*J* = 7 Hz, 1H, 4-H); ^13^C NMR (150 MHz, CD_3_CN, signals with low signal-to-noise ratio were confirmed by HMBC) δ 25.1 (2 × 3’-Me), 39.4 (N^+^Me), 56.3 (C3’), 117.7 (C7’), 121.8 (C3), 121.9 (C4’’), 123.5 (C9’’), 124.4 (C4’), 124.7 (C7), 125.4 (C1), 127.4 (C9a’’), 128.4 (C9), 129.2 (CH), 129.6 (C7’’), 131.1 (C6’), 131.2 (C6’’), 131.4 (C8’’), 132.9 (C5’), 132.9 (CH’), 133.1 (C5a’’), 135.5 (C5’’), 137.5 (C4), 137.6 (C6), 138.5 (C8), 140.0 (C9b’’), 143.6 (C7a’), 144.6 (C9a), 144.6 (C3a’), 145.6 (C2), 148.8 (C3a’’), 151.9 (C2’’), 170.8 (C2’); MS–ESI^+^ (*m*/*z*): 241 (100, M − 2BF_4_), 323 (29, M − C_11_H_12_N − BF_4_), 480 (80, M − HBF_4_ − BF_4_), 568 (89, M − BF_4_).

**(*E*)-2,3,10,11-Tetramethoxy-8-(2-(2-(1,3,3-trimethyl-3*H*-indol-1-ium-2-yl)naphtho[1,2-*d*]oxazol-4-yl)vinyl)coralyne (4b):** ^1^H NMR (600 MHz, CD_3_CN) δ 2.03 (s, 6H, 2 × 3’-Me), 3.97 (s, 3H, OMe), 4.05 (s, 3H, OMe), 4.19 (s, 3H, OMe), 4.21 (s, 3H, OMe), 4.73 (s, 3H, N^+^Me), 7.54 (s, 1H, ar-H), 7.74–7.81 (m, 5H, 5 × ar-H), 7.83–7.85 (m, 2H, 2 × ar-H), 7.89 (ddd, ^3^*J* = 8 Hz, ^3^*J* = 8 Hz, ^4^*J* = 1 Hz, 1H, ar-H), 7.97–7.98 (m, 1H, ar-H), 8.01 (ddd, ^3^*J* = 8 Hz, ^3^*J* = 8 Hz, ^4^*J* = 1 Hz, 1H, ar-H), 8.10 (d, ^3^*J* = 17 Hz, 1H, CH), 8.27 (s, 1H, ar-H), 8.30 (d, ^3^*J* = 8 Hz, 1H, ar-H), 8.69 (s, 1H, ar-H), 8.78–8.79 (m, 1H, ar-H), 8.84 (dd, ^3^*J* = 8 Hz, ^4^*J* = 1 Hz, 1H, ar-H), 9.50 (s, 1H, 13-H); MS–ESI^+^ (*m*/*z*): 350 (62, M − 2BF_4_), 543 (43, M − C_11_H_12_N − BF_4_), 700 (100, M − HBF_4_ − BF_4_), 788 (73, M − BF_4_).

**(*E*)-2-(2-(2-(2-Hydroxy-1,3,3-trimethylindolin-2-yl)naphtho[1,2-*d*]oxazol-4-yl)vinyl)quinolizinium (5):** The solutions that were used for the NMR spectroscopic and mass spectrometric analysis of **4a** were diluted up to half with D_2_O or H_2_O, respectively. The solutions were kept in the dark for 1 h and then used for the analysis of the oxazole derivative **5**. ^1^H NMR (400 MHz, CD_3_CN/D_2_O, 1:1) δ 0.93 (s, 3H, 3’-Me), 1.48 (s, 3H, 3’-Me), 3.23 (s, 3H, NMe), 6.69 (d, ^3^*J* = 8 Hz, 1H, 7’-H), 6.87 (ddd, ^3^*J* = 7 Hz, ^3^*J* = 7 Hz, ^4^*J* = 1 Hz, 1H, 5’-H), 7.12 (dd, ^3^*J* = 7 Hz, ^4^*J* = 1 Hz, 1H, 4’-H), 7.25 (ddd, ^3^*J* = 8 Hz, ^3^*J* = 8 Hz, ^4^*J* = 1 Hz, 1H, 6’-H), 7.45 (d, ^3^*J* = 16 Hz, 1H, CH), 7.62 (ddd, ^3^*J* = 8 Hz, ^3^*J* = 6 Hz, ^4^*J* = 1 Hz, 1H, 7’’-H), 7.73 (ddd, ^3^*J* = 7 Hz, ^3^*J* = 7 Hz, ^4^*J* = 1 Hz, 1H, 8’’-H), 7.84 (ddd, ^3^*J* = 7 Hz, ^3^*J* = 7 Hz, ^4^*J* = 1 Hz, 1H, 7-H), 7.87 (d, ^3^*J* = 16 Hz, 1H, CH’), 8.07–8.14 (m, 4H, 6’’-H, 5’’-H, 3-H, 1-H), 8.21 (ddd, ^3^*J* = 7 Hz, ^3^*J* = 7 Hz, ^4^*J* = 1 Hz, 1H, 8-H), 8.29 (d, ^3^*J* = 8 Hz, 1H, 9-H), 8.41 (d, ^3^*J* = 8 Hz, 1H, 9’’-H), 8.92 (d, ^3^*J* = 8 Hz, 2H, 4-H, 6-H); MS–ESI^+^ (*m*/*z*): 323 (100, M − C_11_H_13_NO − BF_4_), 498 (24, M − BF_4_).

## Supporting Information

File 1 Experimental procedures, additional spectroscopic data, ^1^H NMR and ^13^C NMR spectra.
