# Supplementary material for: Cation-induced ring-opening and oxidation reaction of photoreluctant spirooxazine–quinolizinium conjugates
Source: Beilstein J Org Chem. 2020 May 5;16:904–16. doi: 10.3762/bjoc.16.82 (PMC7214873; doi:10.3762/bjoc.16.82)
Supplement: File 1 — Experimental procedures, additional spectroscopic data, 1H NMR and 13C NMR spectra. [file Beilstein_J_Org_Chem-16-904-s001.pdf]

BEILSTEIN JOURNAL OF ORGANIC CHEMISTRY

Supporting Information

for

Cation-induced ring-opening and oxidation reaction of photoreluctant spirooxazine–quinolizinium conjugates

Phil M. Pithan, Sören Steup and Heiko Ihmels

Beilstein J. Org. Chem. **2020,** *16,* 904–916. doi:10.3762/bjoc.16.82

Experimental procedures, additional spectroscopic data, ^{1}H NMR and ^{13}C NMR spectra

License and Terms: This is a supporting information file under the terms of the Creative Commons Attribution License (http://creativecommons.org/licenses/by/4.0). Please note that the reuse, redistribution and reproduction in particular requires that the authors and source are credited.
The license is subject to the *Beilstein Journal of Organic Chemistry* terms and conditions: (https://www.beilstein-journals.org/bjoc)

Table of Contents

1 Experimental Section **S2**

1.1 Methods S2

1.2 Synthesis S2

1.2.1 Synthesis of the 5'-formyl-substituted spirooxazine **1b** S2

2 Additional spectroscopic data **S5**

3 ^{1}H and ^{13}C NMR spectra **S8**

4 References **S18**

1 Experimental Section

1.1 Methods

The relative fluorescence quantum yields of the derivatives **3a**, **3b**, and **4a** were determined under identical conditions, i.e., the same cuvettes were used, and the measurements were performed at a constant temperature with the same settings on the spectrometer, such as detection wavelength, excitation wavelength, detector voltage, slit bandwidths (5 nm), and collection rate (120 nm·min^{-1}). Coumarin 307 (Φ_{fl} = 0.58 in MeCN)[1] and Rhodamine 6G (Φ_{fl} = 0.95 in EtOH)[2] were used as standards. The emission spectra were collected from diluted solutions with Abs < 0.10 at the excitation wavelength. The emission spectra were smoothed with the implemented moving-average function by a factor of 5. After integration of the fluorescence band, the relative fluorescence quantum yields were calculated according to Equation S1.[3]

$$\phi_{fl} = \frac{J_x \cdot (1 - T_S)}{J_S \cdot (1 - T_x)} \cdot \frac{n_x^2}{n_S^2} \cdot \phi_{fl,S} \qquad \text{(Eq. S1)}$$

The subscripts "x" and "s" refer to the substance under investigation and a reference compound, respectively; $J = \int I_{fl}(\lambda)d\lambda$ is the emission integral over the area of interest; T is the optical transmittance of the sample solution at the excitation wavelength, λ_{ex}; n is the refractive index of the sample or standard solution.

1.2 Synthesis

1.2.1 Synthesis of the 5'-formyl-substituted spirooxazine **1b**

The known 5'-formyl-substituted spirooxazine **1b**[4] was synthesized by the condensation of 3-(hydroxymethyl)-1-nitroso-2-naphthol (**6**)[5] with 1,3,3-trimethyl-2-methyleneindoline and subsequent oxidation of the alcohol **1c** with Dess–Martin periodinane (Scheme S1). As the ^{13}C NMR data for the spirooxazine **1b** have not been reported in the original literature, they will be presented herein.

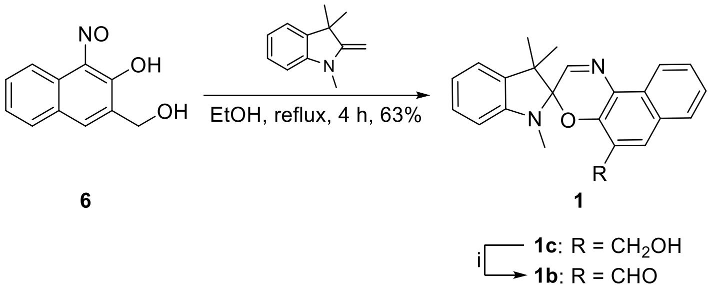

Scheme S1: Synthesis of the 5'-formyl-substituted spirooxazine **1b**; reagents and reaction conditions: (i) Dess–Martin periodinane, CH_2Cl_2, rt, 1 h, 51%.

5’-Hydroxymethyl-1,3,3-trimethylspiro[indoline-2-3’-naphtho[2,1-*b*][1,4]oxazine] (**1c**)

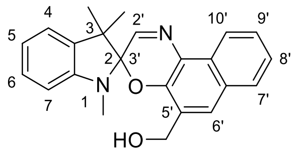

To a solution of 3-(hydroxymethyl)-1-nitroso-2-naphthol (**4**[5], 4.00 g, 19.7 mmol) in ketone-free EtOH (200 mL) was added freshly distilled 1,3,3-trimethyl-2-methyleneindoline (3.76 g, 21.7 mmol, 3.8 mL) at 75 °C, and the reaction mixture was stirred under reflux for 4 h. After cooling to rt, the solvent was removed under reduced pressure. The product **1c** was isolated by flash column chromatography (SiO_2; eluent: *n*-hexane/EtOAc 8/2, R_f = 0.37), crystallized from EtOH, and obtained as green, crystalline solid (4.43 g, 12.4 mmol, 63%, lit.: 55%[4a]; mp = 141–143 °C (lit.: 141 °C)[4a] – ^{1}H NMR (600 MHz, $CDCl_3$): δ = 1.37 (s, 3 H, 3-CH_3), 1.39 (s, 3 H, 3-CH_3), 2.02 (t, ^3J = 6 Hz, 1 H, OH), 2.73 (s, 3 H, N-CH_3), 4.64 (dd, ^2J = 14 Hz, ^3J = 6 Hz, 1 H, 5’-CH-H), 4.74 (dd, ^2J = 14 Hz, ^3J = 5 Hz, 1 H, 5’-CH-H’), 6.57 (d, ^3J = 8 Hz, 1 H, 7-H), 6.91 (ddd, ^3J = 8 Hz, ^3J = 8 Hz, ^4J = 1 Hz, 1 H, 5-H), 7.10 (dd, ^3J = 7 Hz, ^4J = 1 Hz, 1 H, 4-H), 7.22 (ddd, ^3J = 9 Hz, ^3J = 8 Hz, ^4J = 1 Hz, 1 H, 6-H), 7.43 (ddd, ^3J = 8 Hz, ^3J = 7 Hz, ^4J = 1 Hz, 1 H, 8’-H), 7.59 (ddd, ^3J = 8 Hz, ^3J = 7 Hz, ^4J = 1 Hz, 1 H, 9’-H), 7.72 (s, 1 H, 6’-H), 7.77 (d, ^3J = 8 Hz, 1 H, 7’-H), 7.80 (s, 1 H, 2’-H), 8.55 (d, ^3J = 8 Hz, 1 H, 10’-H). – ^{13}C NMR (150 MHz, $CDCl_3$): δ = 20.9 (3-Me), 25.4 (3-Me), 29.6 (NMe), 51.5 (C3), 61.1 (5’-CH_2OH), 98.6 (C2/C3’), 107.2 (C7), 120.0 (C5), 121.4 (C5’), 121.5 (C4), 122.9 (C10b’), 124.5 (C8’), 126.9 (C9’), 127.8 (C7’), 127.9 (C6’), 128.1 (C10’), 128.2 (C6), 128.8 (C6a’), 130.3 (C10a’), 135.7 (C3a), 142.1 (C4a’), 147.1 (C7a), 150.4 (C2’).

5’-Formyl-1,3,3-trimethylspiro[indoline-2-3’-naphtho[2,1-*b*][1,4]oxazine] (**1b**)

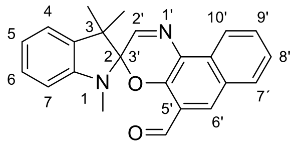

To a solution of the alcohol **1c** (1.10 g, 3.07 mmol) in anhydrous CH_2Cl_2 (90 mL) was added Dess–Martin periodinane (1.56 g, 3.68 mmol) under an argon atmosphere. The solution was stirred for 1 h at rt, washed with saturated $NaHCO_3$ solution (2 × 40 mL), dried with Na_2SO_4, and filtered from the drying agent. The solvent was removed under removed pressure. The product **1b** was isolated by flash column chromatography (SiO_2; eluent: *n*-hexane/EtOAc 85/15, R_f = 0.48), crystallized from EtOAc/*n*-hexane at −25 °C, and obtained as yellow, amorphous solid (555 mg, 1.56 mmol, 51%, lit.: 65–67%[4b,4c]); mp = 182–184 °C (lit.: 176 °C[4b]). – ^{1}H NMR (400 MHz, $CDCl_3$): δ = 1.40 (s, 3 H, 3-CH_3), 1.41 (s, 3 H, 3-CH_3), 2.81 (s, 3 H, N-CH_3), 6.60 (d, ^3J = 8 Hz, 1 H, 7-H), 6.92 (ddd, ^3J = 8 Hz, ^3J = 8 Hz, ^4J = 1 Hz, 1 H, 5-H), 7.10 (dd, ^3J = 7 Hz, ^4J = 1 Hz, 1 H, 4-H), 7.23 (ddd, ^3J = 9 Hz, ^3J = 8 Hz, ^4J = 1 Hz, 1 H, 6-H), 7.46 (ddd, ^3J = 8 Hz, ^3J = 7 Hz, ^4J = 1 Hz, 1 H, 8’-H), 7.69 (ddd, ^3J = 9 Hz, ^3J = 7 Hz, ^4J = 1 Hz, 1 H, 9’-H), 7.86 (s, 1 H, 2’-H), 7.89 (d, ^3J = 8 Hz, 1 H, 7’-H), 8.32 (s, 1 H, 6’-H), 8.58

(d, ^3J = 9 Hz, 1 H, 10'-H), 10.36 (s, 1 H, 5'-CHO). – ^{13}C NMR (100 MHz, $CDCl_3$): δ = 21.2 (3-Me), 25.4 (3-Me), 29.6 (NMe), 52.0 (C3), 98.9 (C2/C3'), 107.2 (C7), 120.2 (C5), 121.4 (C4), 121.8 (C10'), 123.2 (C5'), 123.7 (C10b'), 125.3 (C8'), 127.9 (C6a'), 128.1 (C6), 129.9 (C9'), 130.1 (C6'), 130.1 (C7'), 133.8 (C10a'), 135.4 (C3a), 144.5 (C4a'), 147.1 (C7a), 151.8 (C2'), 188.6 (5'-CHO).

2 Additional spectroscopic data

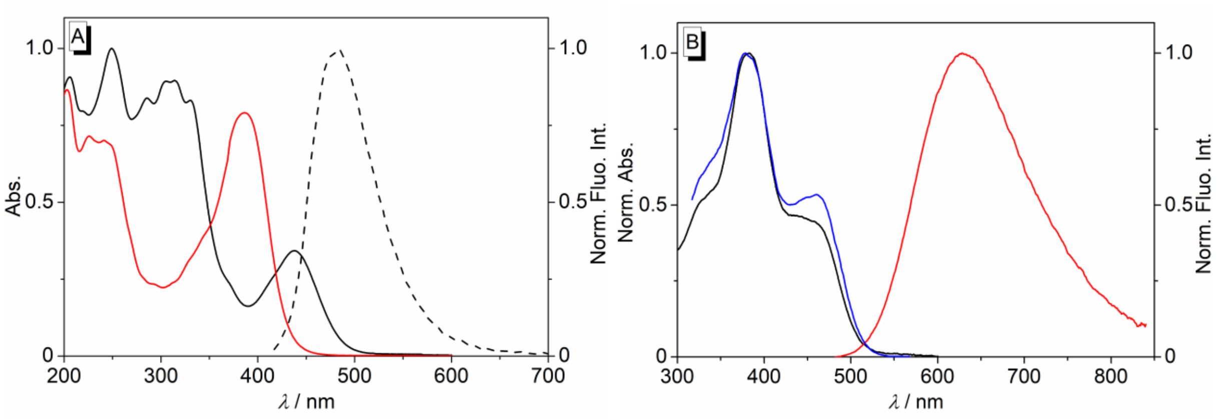

Figure S1: A: Absorption spectra (c = 20 µM in MeCN, solid lines) of the derivatives **3a** (red) and **3b** (black) and normalized emission spectrum of **3b** (Abs = 0.10 at λ_{ex} = 400 nm, dashed line). B: Normalized absorption (c = 20 µM, black), emission (c = 5 µM, λ_{ex} = 470 nm, red), and fluorescence excitation spectrum (c = 5 µM, λ_{em} = 600 nm, blue) of **4a** in MeCN.

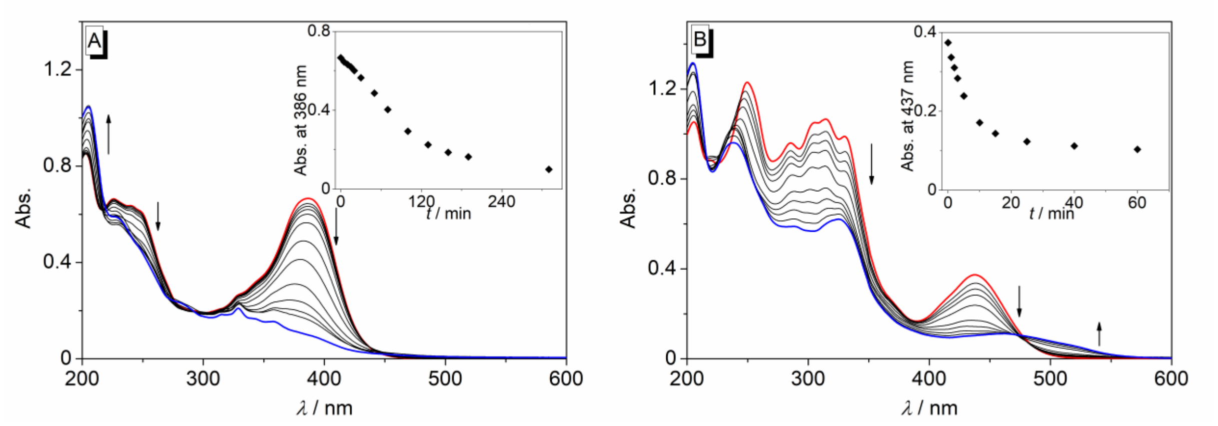

Figure S2: Spectral changes during the irradiation of **3a** (c = 17 µM) (A) and **3b** (c = 20 µM) (B) in MeCN with 420 nm light. The arrows indicate the changes of absorption upon irradiation. Red: Spectra of the pure ligand solutions; blue: spectra at the end of the irradiation. Insets: Plot of the absorption vs the irradiation time t.

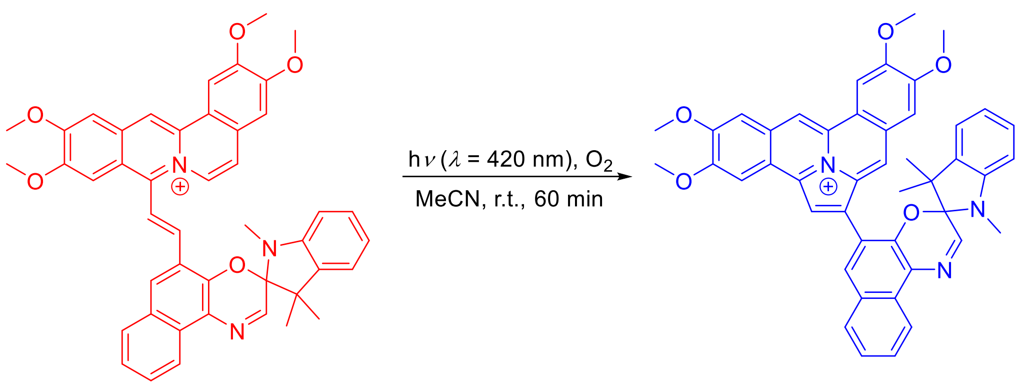

3b **7**

Scheme S2: Formation of the pyrroloquinolizinium derivative **7** by irradiation of **3b** (cf. Figure S2B).

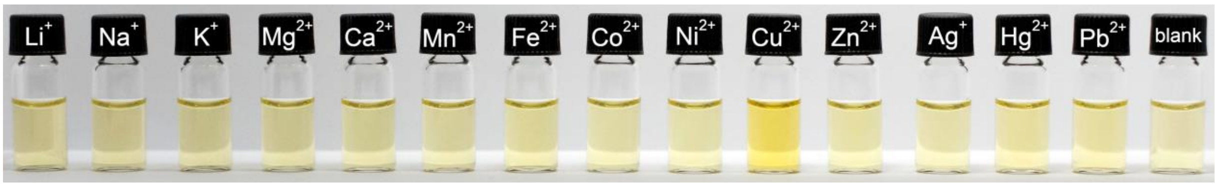

Figure S3: Colors of solutions resulting from the addition of metal ions (*c* = 50 µM) to derivative **3a** (*c* = 20 µM in MeCN); *t* = 1 h.

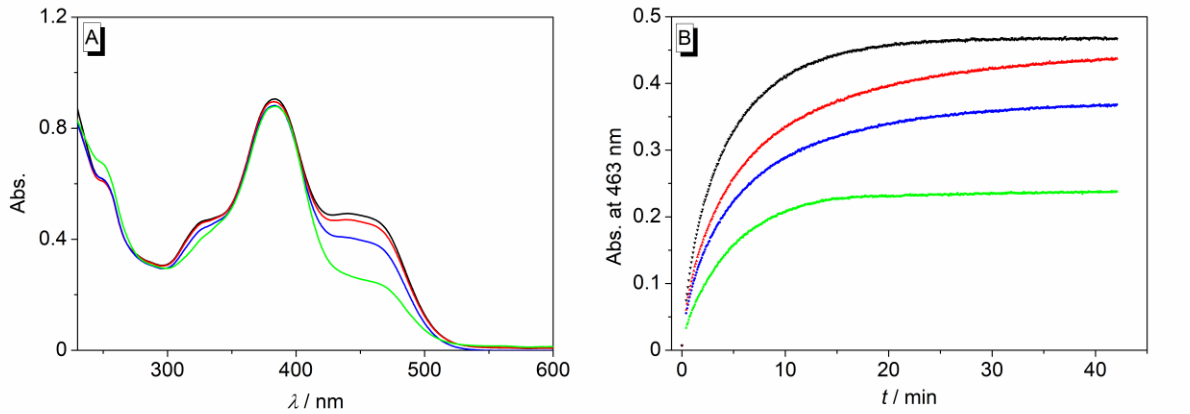

Figure S4: A: Absorption spectra of **3a** in MeCN (*c* = 20 µM) 40 min after the addition of 1.0 (green), 1.5 (blue), 2.0 (red) and 3.0 equiv (black) of Cu^{2+}. B: Plot of the absorbance at 463 nm vs the time *t* after the addition of Cu^{2+}.

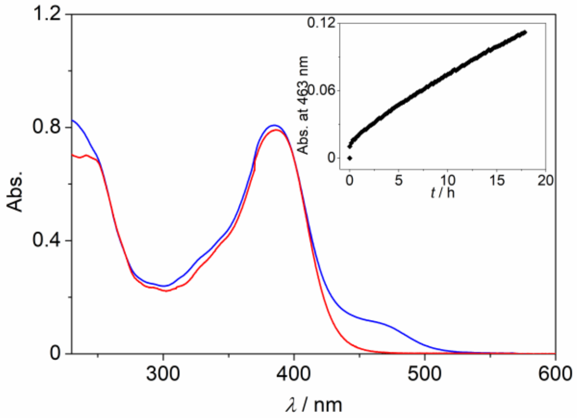

Figure S5: A: Absorption spectra of **3a** in MeCN (*c* = 20 µM) in the absence (red) and in the presence of 3.0 equiv Hg^{2+} ca. 18 h after the addition (blue). Inset: Plot of the absorbance at 463 nm vs the time *t* after the addition of Hg^{2+}.

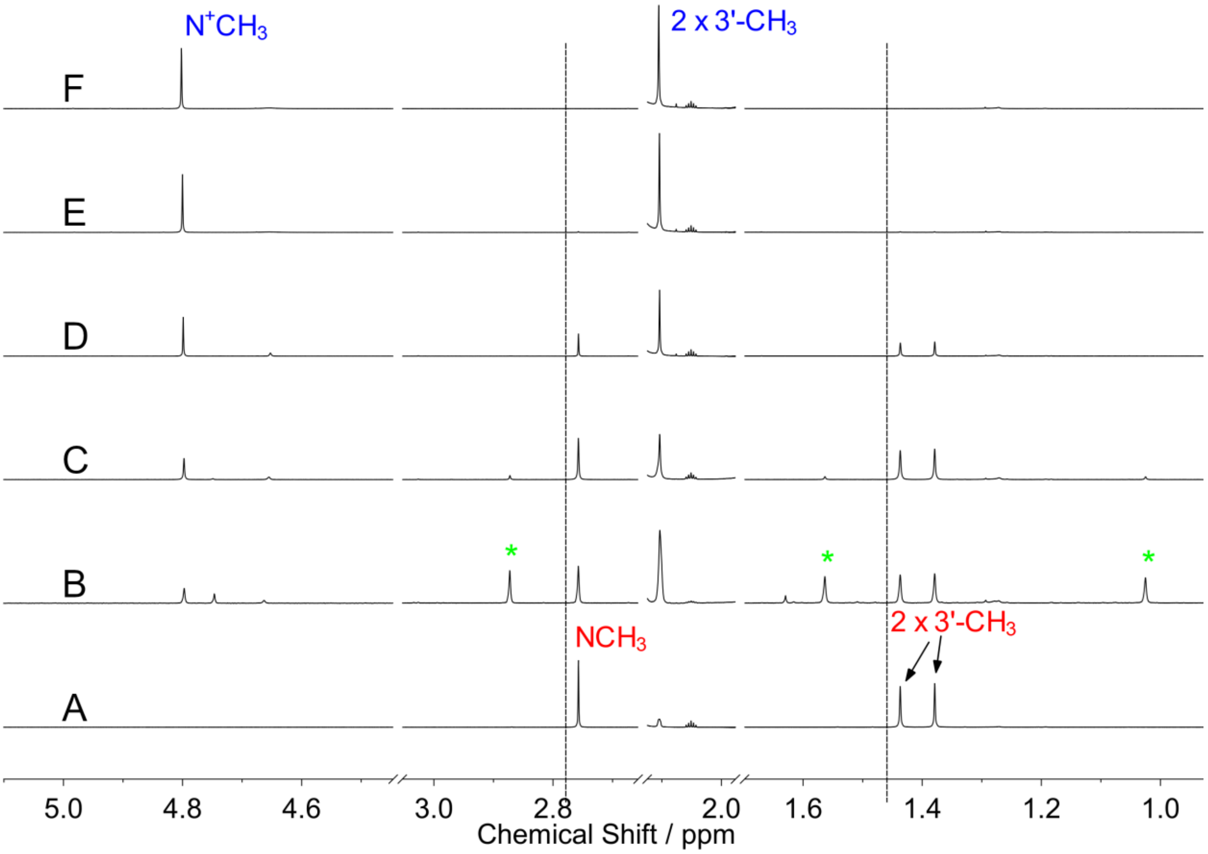

Figure S6: ^{1}H NMR spectra (600 MHz, 0.8–5.2 ppm) of **3a** (*c* = 2.0 mM) in the absence (A) and in the presence (B–F) of Cu^{2+} (B: 0.50 mM, C: 1.0 mM, D: 2.0 mM, E: 3.0 mM, F: 4.0 mM) in CD_3CN (cf. Scheme 2).

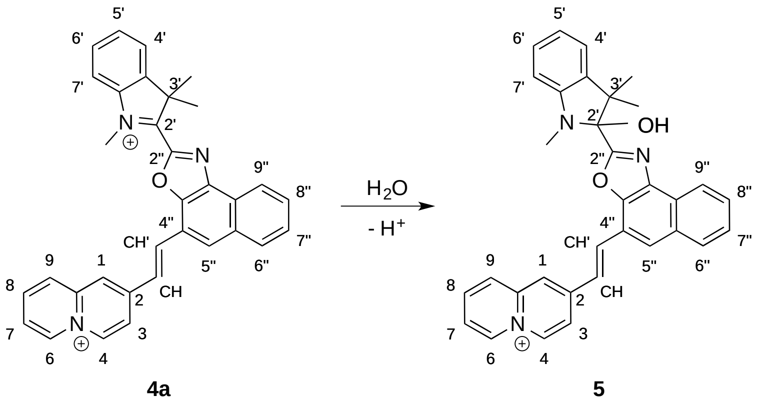

Scheme S3: Formation of the 2'-hydroxy-substituted oxazole derivative **5**.

3 ^{1}H and ^{13}C NMR spectra

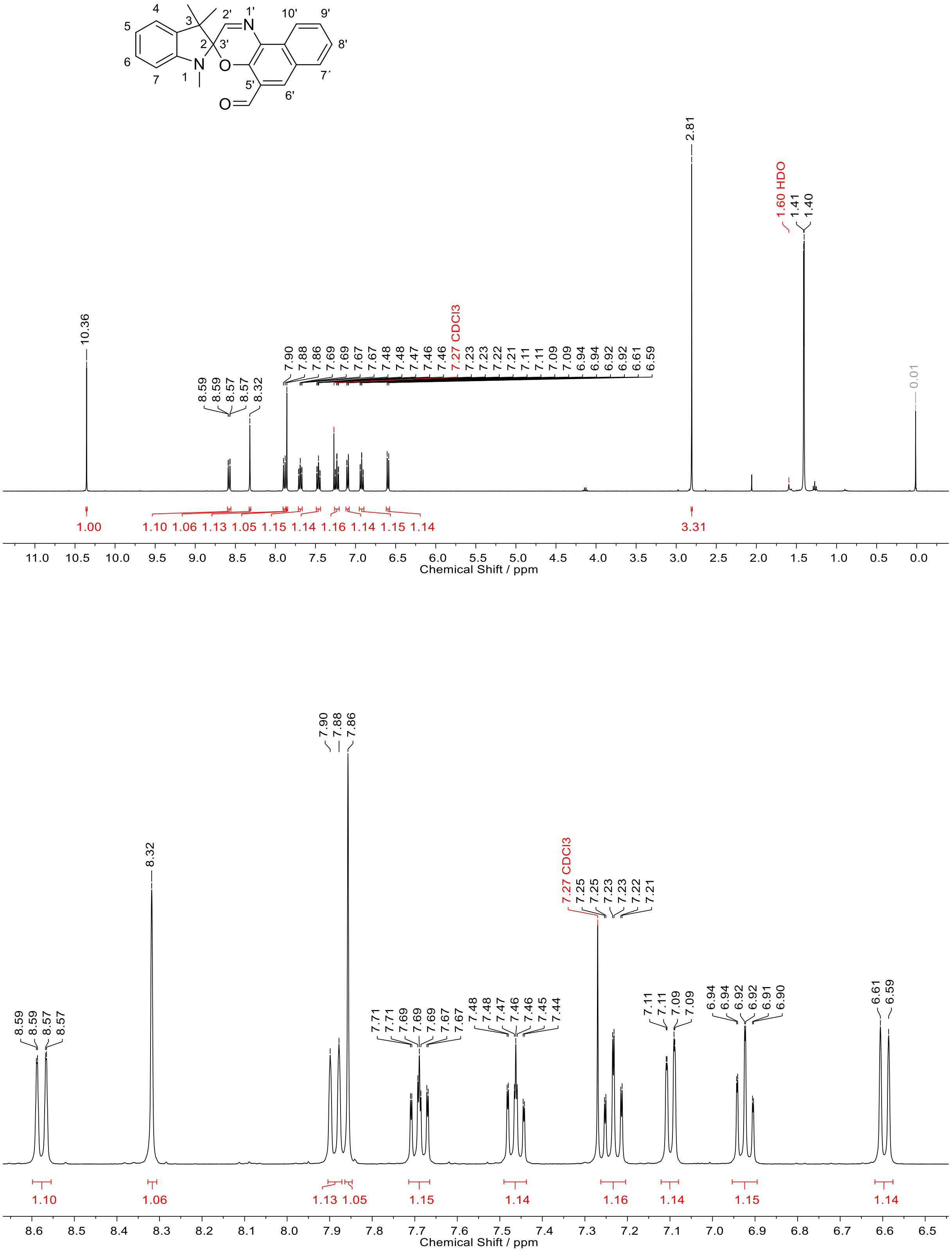

Figure S7: ^{1}H NMR spectrum (400 MHz) of derivative **1b** in $CDCl_3$ (top) with expansion (bottom).

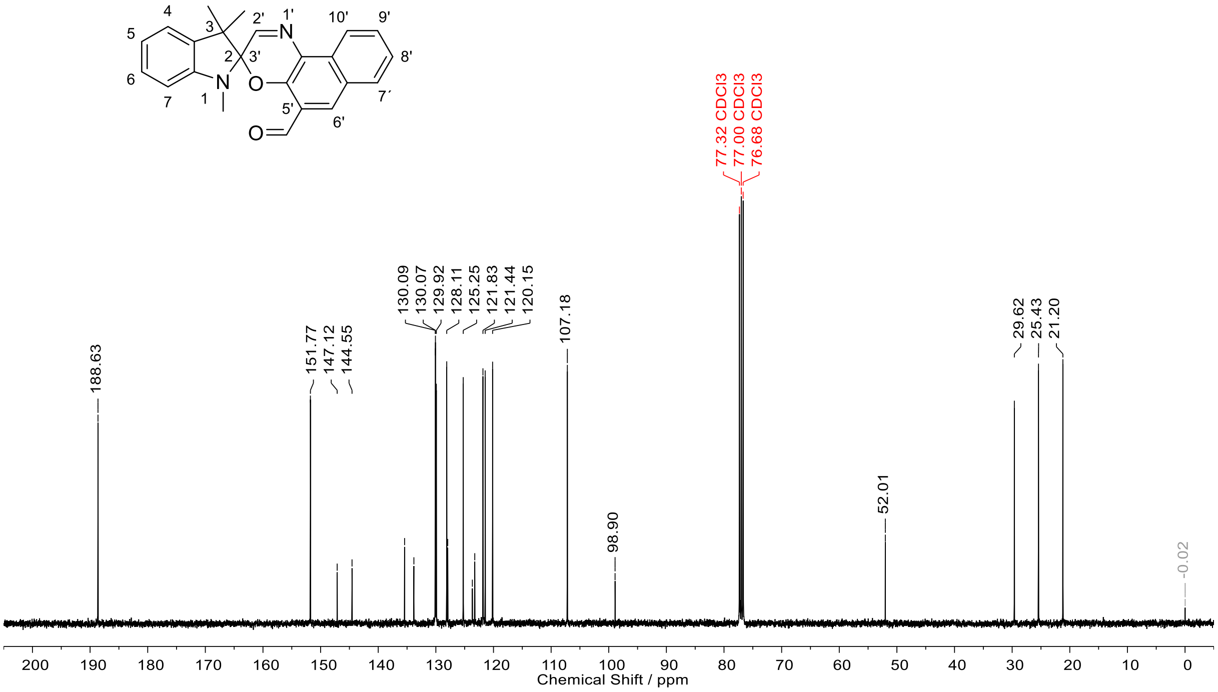

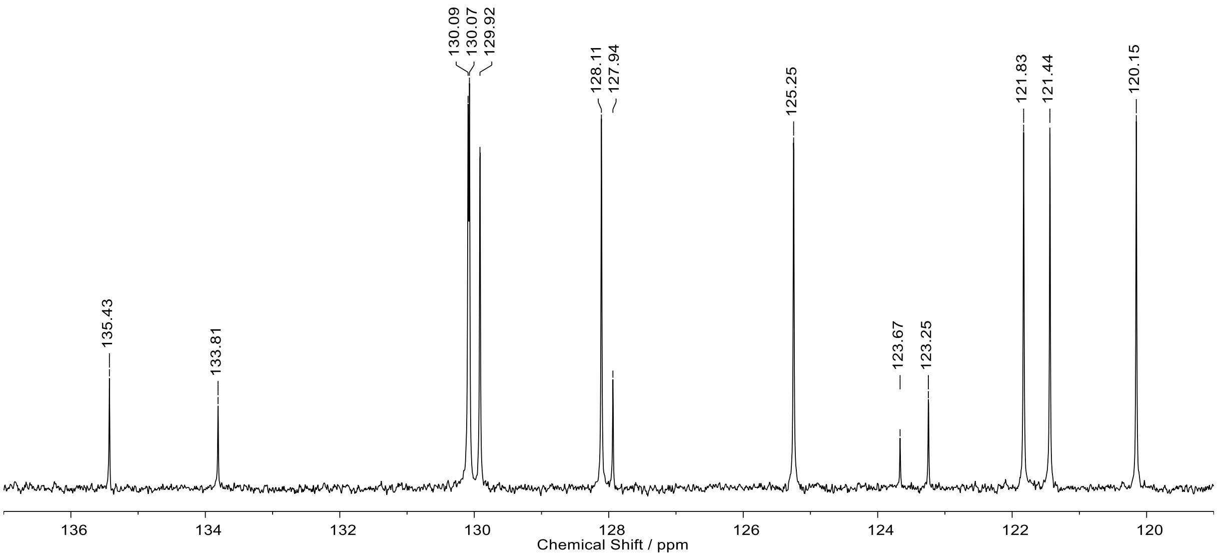

Figure S8: ^{13}C NMR spectrum (100 MHz) of derivative **1b** in $CDCl_3$ (top) with expansion (bottom).

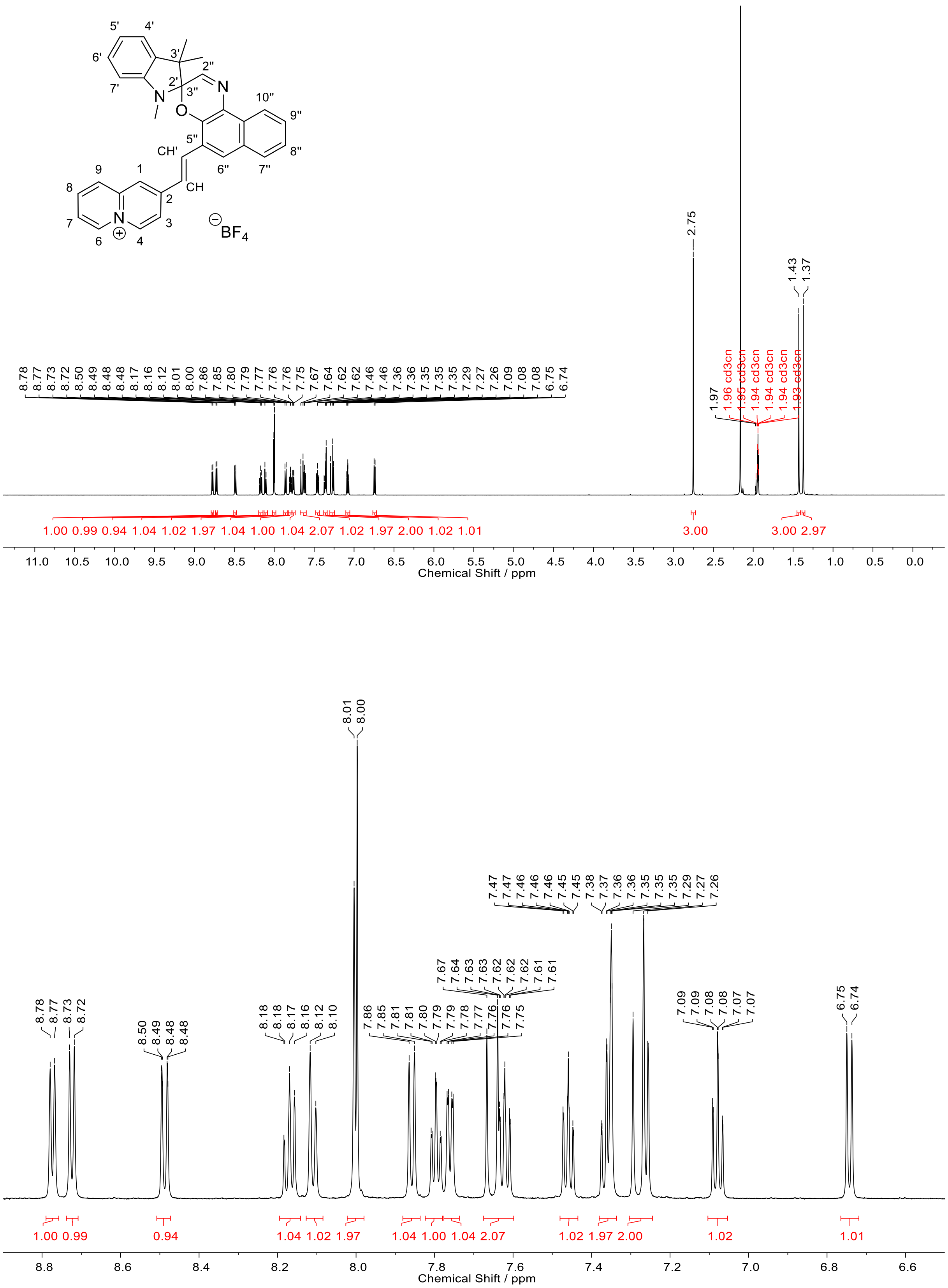

Figure S9: ^{1}H NMR spectrum (500 MHz) of derivative **3a** in CD_3CN (top) with expansion (bottom).

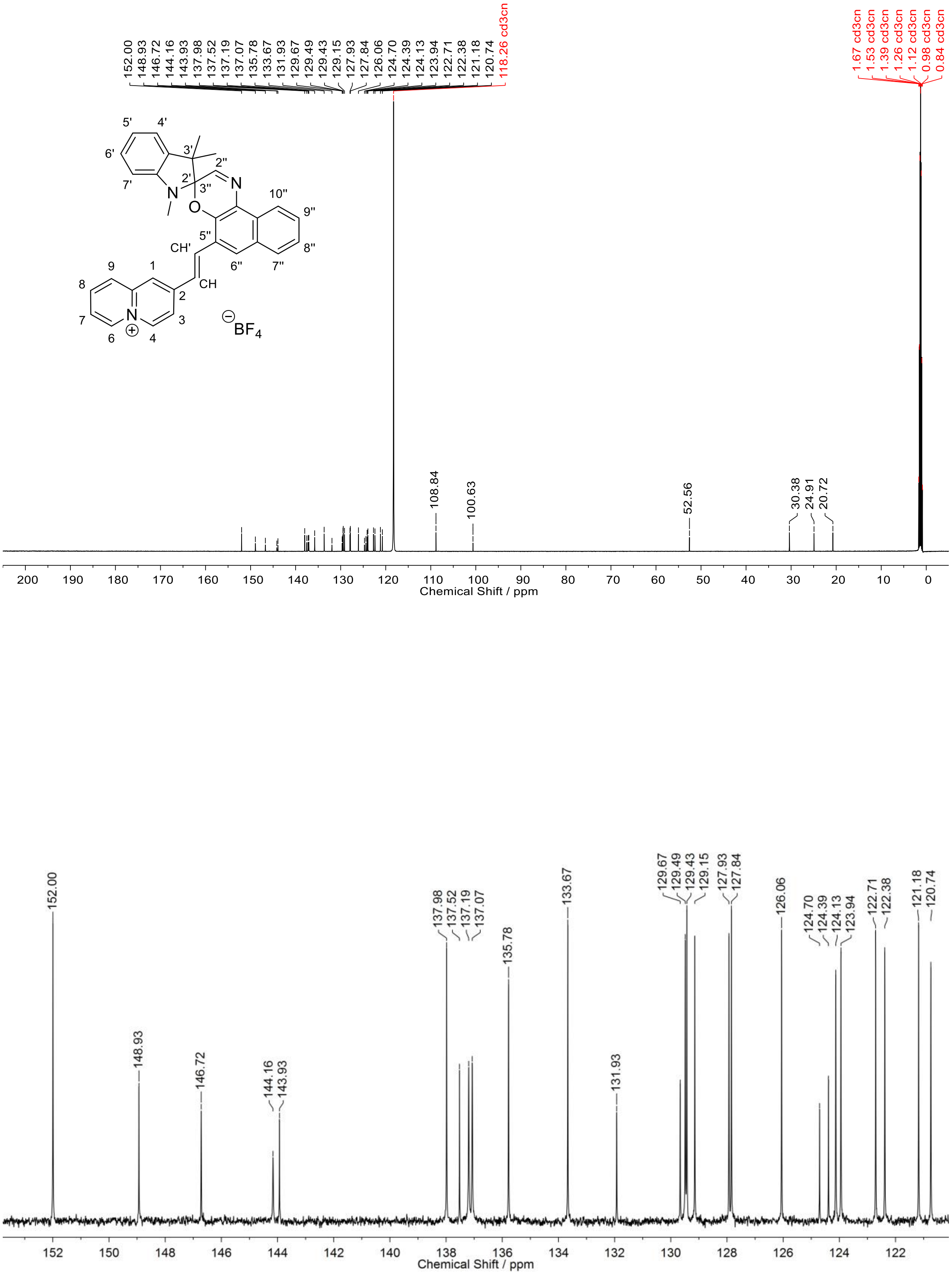

Figure S10: ^{13}C NMR spectrum (125 MHz) of derivative **3a** in CD_3CN (top) with expansion (bottom).

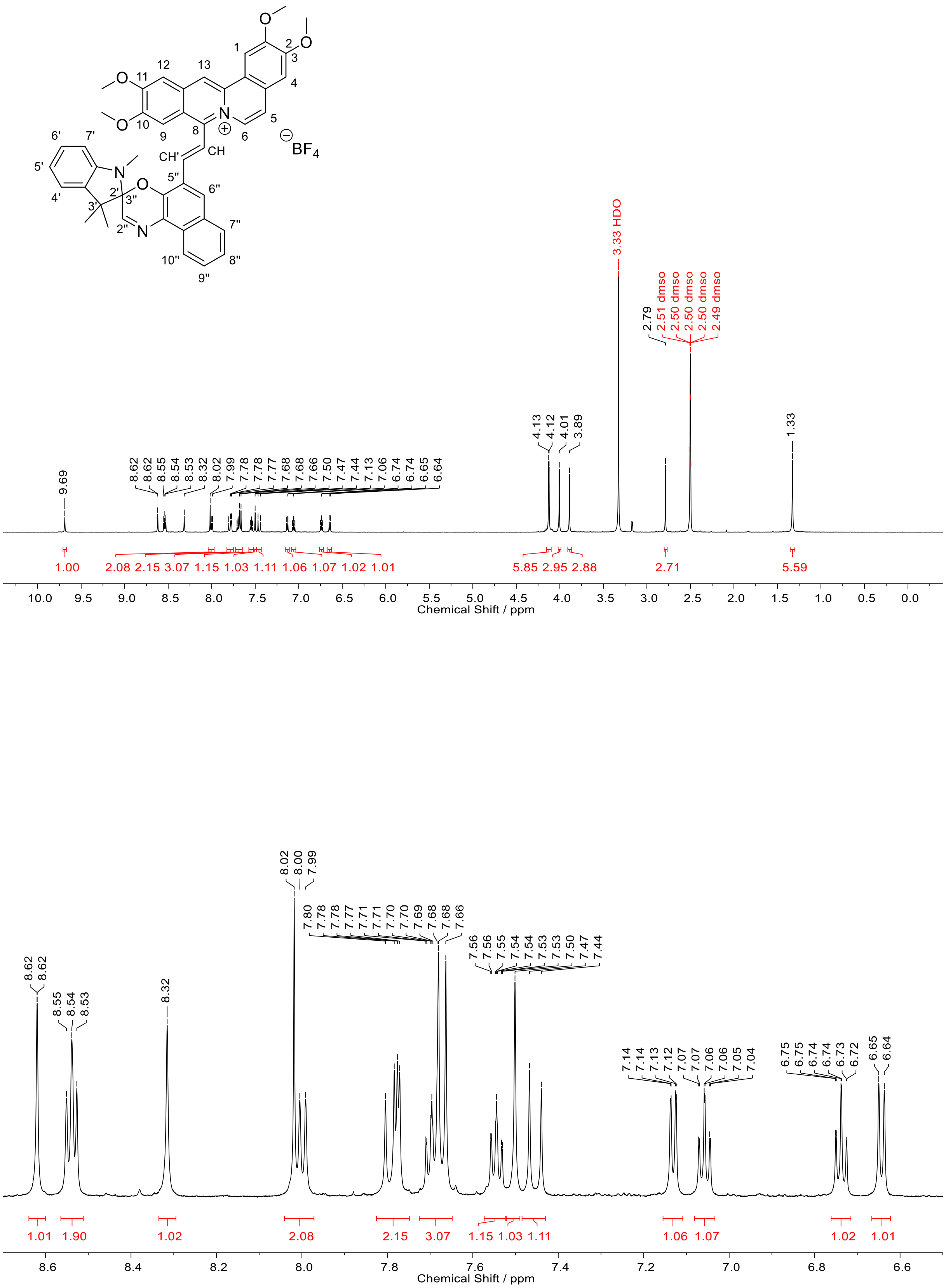

Figure S11: ^{1}H NMR spectrum (600 MHz) of derivative **3b** in DMSO-d_6 (top) with expansion (bottom).

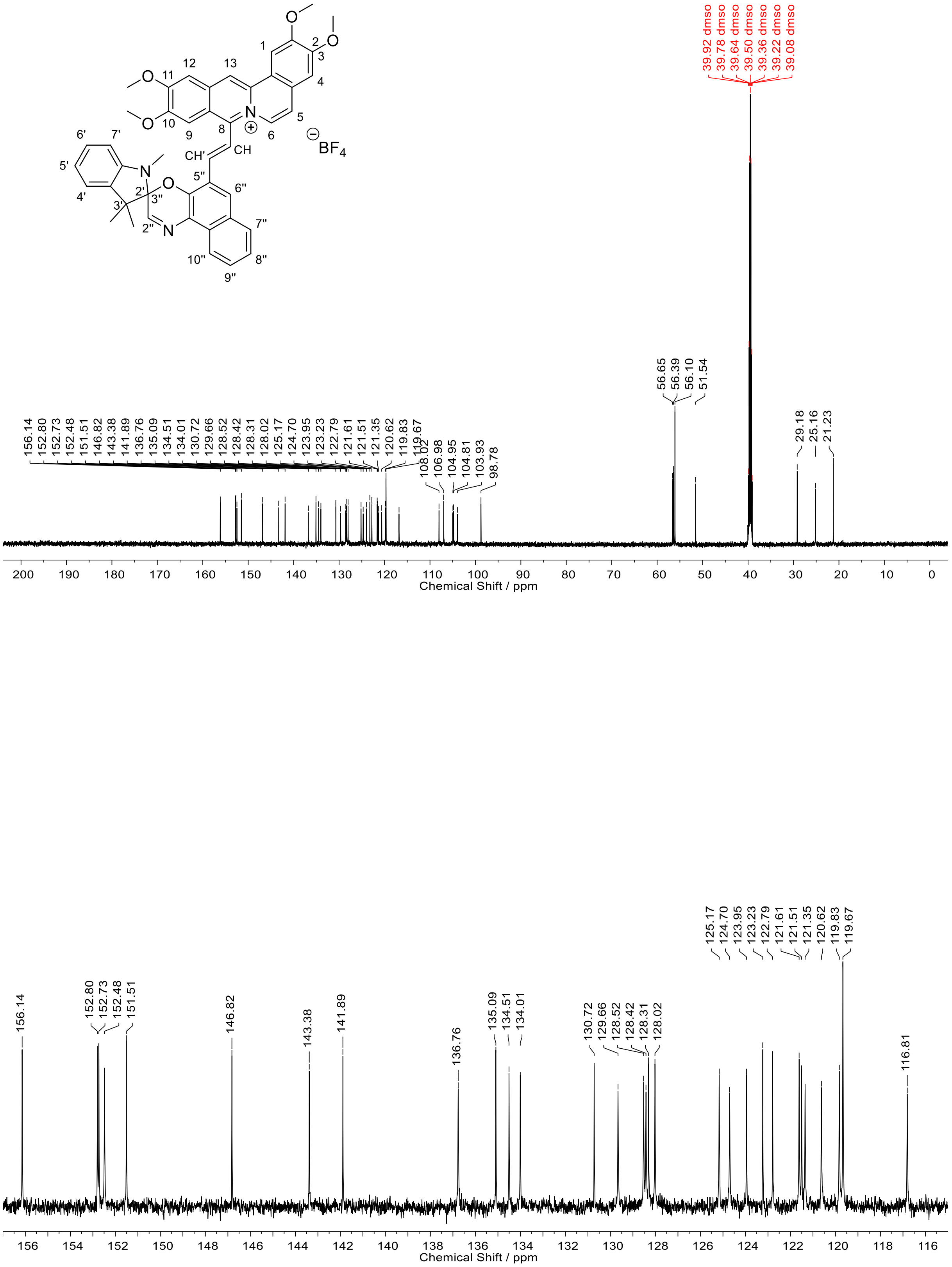

Figure S12: ^{13}C NMR spectrum (150 MHz) of derivative **3b** in DMSO-d_6 (top) with expansion (bottom).

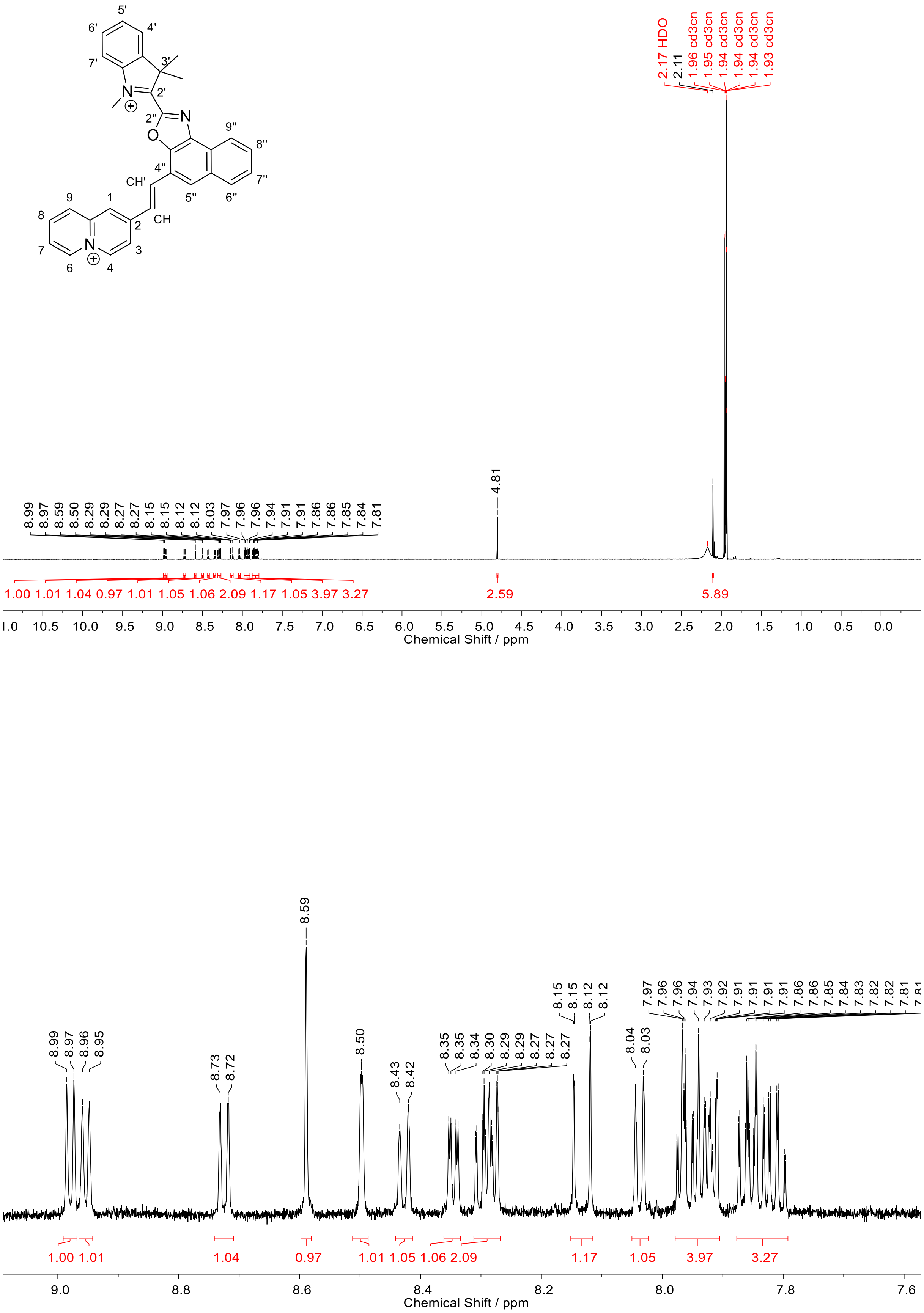

Figure S13: ^{1}H NMR spectrum (600 MHz) of derivative **4a** in CD_3CN (top) with expansion (bottom).

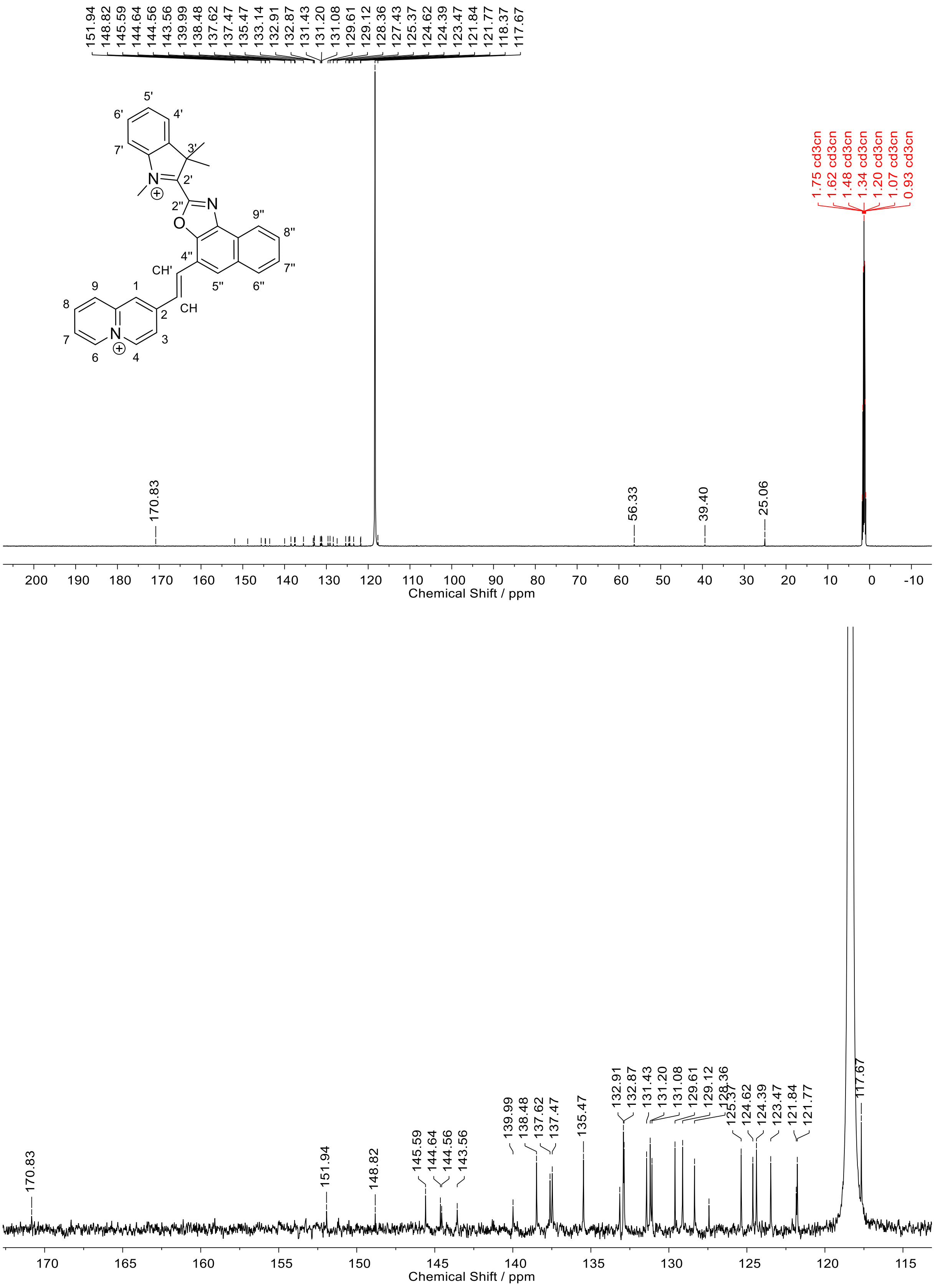

Figure S14: ^{13}C NMR spectrum (150 MHz) of derivative **4a** in CD_3CN (top) with expansion (bottom).

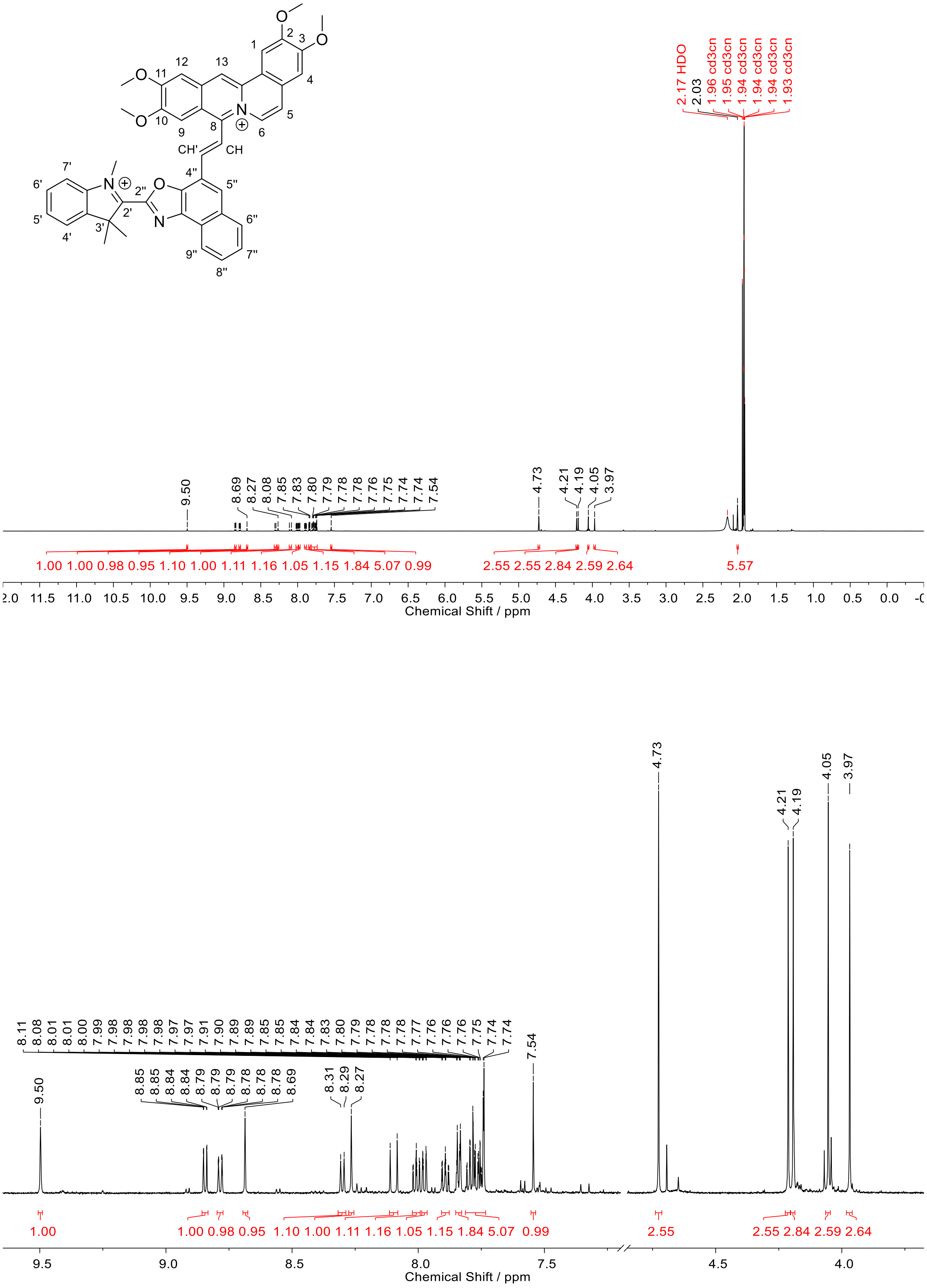

Figure S15: ^{1}H NMR spectrum (600 MHz) of derivative **4b** in CD_3CN (top) with expansion (bottom).

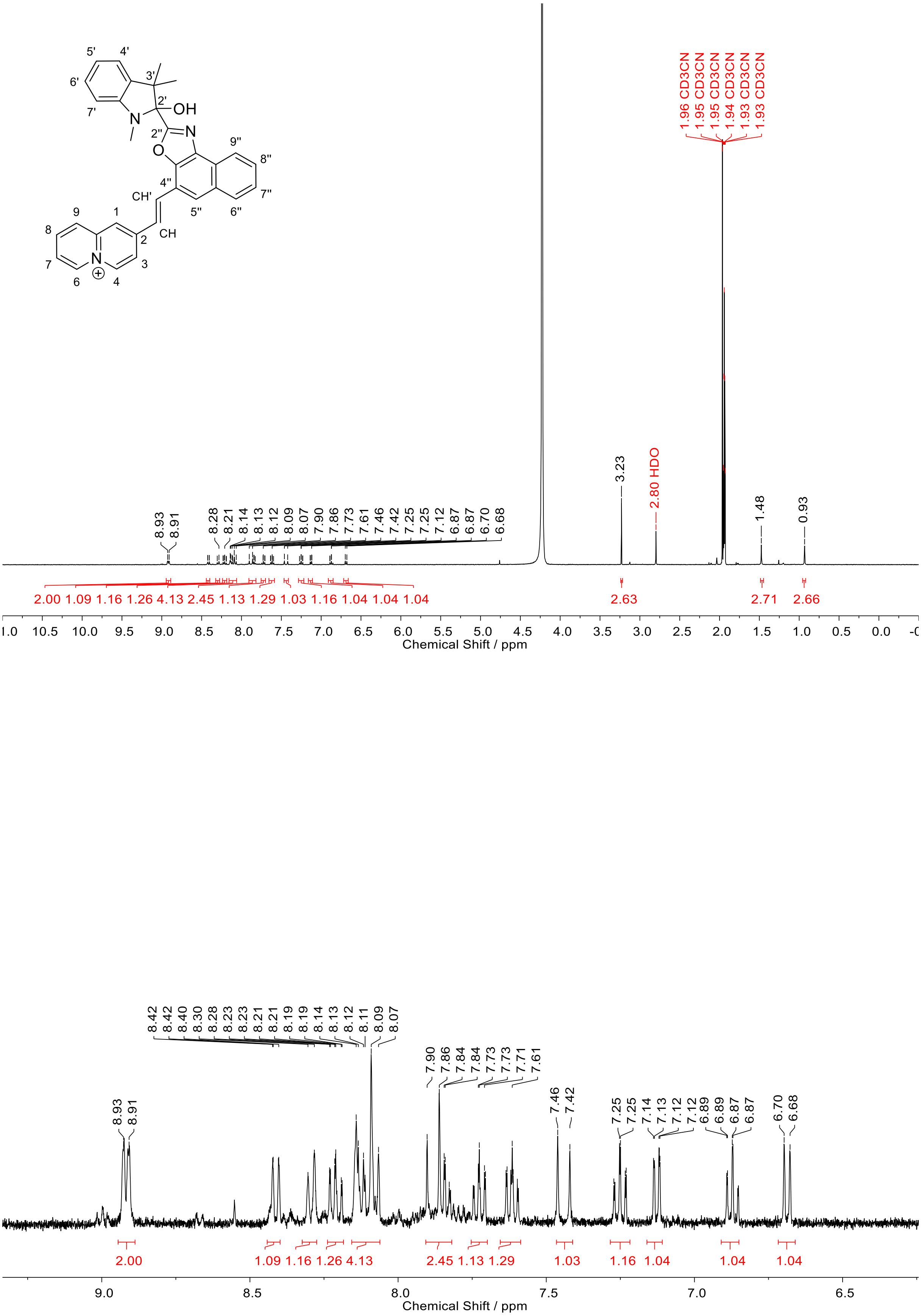

Figure S16: ^{1}H NMR spectrum (400 MHz) of derivative **5** in CD_3CN/D_2O 1/1 (top) with expansion (bottom).

4 References

[1] G. Jones, W. R. Jackson, C. Y. Choi, W. R. Bergmark, *J. Phys. Chem.* **1985**, *89*, 294–300.

[2] D. Magde, R. Wong, P. G. Seybold, *Chem. Phys. Lett.* **2002**, *75*, 327–334.

[3] a) G. A. Crosby, J. N. Demas, *J. Phys. Chem.* **1971**, *75*, 991–1024; b) B. Valeur, M. N. Berberan-Santos, *Molecular Fluorescence*. *Principles and Applications*, Wiley-VCH, Weinheim, **2012**.

[4] a) A. Samat, V. Lokshin, K. Chamontin, D. Levi, G. Pepe, R. Guglielmetti, *Tetrahedron* **2001**, *57*, 7349–7359; b) V. Lokshin, K. Chamontin, R. Guglielmetti, A. Samat, U.S. Patent 6,019,914, **2000**; c) M. Campredon, R. Guglielmetti, B. Luccioni-Houzé, G. Pèpe, A. Alberti, D. Macciantelli, *Free Radical Res.* **1997**, *26*, 529–536.

[5] a) L. E. Miller, W. W. Hanneman, W. L. St. John, R. R. Smeby, *J. Am. Chem. Soc.* **1954**, *76*, 296–297; b) P. E. Georghiou, M. Ashram, H. J. Clase, J. N. Bridson, *J. Org. Chem.* **1998**, *63*, 1819–1826; c) K. Chamontin, V. Lokshin, A. Samat, R. Guglielmetti, R. Dubest, J. Aubard, *Dyes Pigm.* **1999**, *43*, 119–125.
